# Projecting National-Level Prevalence of General Obesity and Abdominal Obesity Among Chinese Adults With Aging Effects

**DOI:** 10.3389/fendo.2022.849392

**Published:** 2022-03-08

**Authors:** Xu Tian, Hui Wang

**Affiliations:** ^1^College of Economics and Management, Academy of Global Food Economics and Policy, China Agricultural University, Beijing, China; ^2^Department of Maternal and Child Health, School of Public Health, Peking University Health Science Center, Beijing, China

**Keywords:** population aging, general obesity, abdominal obesity, projection, sex

## Abstract

**Objectives:**

To explore the impact of population aging on the projected prevalence of obesity among Chinese adults in 2030.

**Methods:**

In total, 71450 observations were extracted from the China Health and Nutrition Survey between 1991 and 2015.Population was projected to 2030 using a Bayesian hierarchical modeling method. Two different approaches were adopted to estimate and project the national prevalence of overweight/obesity from 1991 to 2030. One method assumed a constant population at the base year, while the other allowed the age and gender distributions vary in each year.

**Results:**

Our projection indicated that approximately two-thirds of Chinese adults would be affected by overweight/general obesity in 2030, and more than 60% of Chinese adults will suffer from abdominal obesity in 2030. Ignoring population aging led to an underestimation of overweight, general obesity and abdominal obesity for women by 3.81, 0.06, and 3.16 percentage points (pp), and overweight and abdominal obesity among men by 1.67 and 0.53 pp, respectively; but the prevalence of general obesity among men will be overestimated by 2.11 pp. Similar underestimations were detected in the estimation from 1991 to 2015.

**Conclusions:**

Estimating and projecting the national prevalence of obesity using a constant population structure at the base line would cause significant underestimation if countries are undergoing rapid population aging.

## Highlights

What is already known about this subject?

- China is undergoing a pandemic of obesity and aging.
- The prevalence of obesity in the older population is higher than that in the younger population.

What are the new findings in your manuscript?

- Ignoring population aging led to an underestimation of overweight and abdominal obesity for both women and men in 2030.
- Approximately two-thirds of Chinese adults will be affected by overweight/general obesity in 2030, and more than 60% of Chinese adults will suffer from abdominal obesity in 2030.

How might your results change the direction of research or the focus of clinical practice?

- Prevention of obesity is part of the job of doctors, and guiding patients to establish a healthful lifestyle when they are young is much more important than ever before.

## Introduction

The growing overweight and obesity epidemic has been well documented in both developed countries and developing countries (1–7). The global prevalence of obesity in adults has almost tripled between 1975 and 2016 (8), and the global deaths and disability-adjusted life years (DALYs) attributable to high body mass index (BMI) have more than doubled from 1990 to 2017 (9). In 2017, high BMI was associated with 4.7 million deaths and 147.7 million DALYs globally, and has become an important contributor to the global disease burden (9), particularly for cardiovascular disease (10), type 2 diabetes (11) and certain types of cancers (12).

In China, BMI, waist circumference (WC) and the prevalence of overweight/obesity have been increasing steadily since the early 1980s along with rapid economic development (4, 13–17). In 2020, 34.3% and 16.4% of Chinese adults were affected by overweight and obesity, respectively, indicating that 1 in 2 adults were either overweight or obese (18). Meanwhile, the prevalence of abdominal obesity (waist circumference ≥ 90 in men and ≥85 cm in women respectively) among Chinese adults grew to 46.9% in 2015 (17). The rapid rise of obesity has become a major public health problem in China. The estimated attribution percentage of overweight and obesity-associated noncommunicable disease (NCD) deaths increased from 5.7% in 1990 to 11.1% in 2019 in China (19).

Previous literature has documented that the increasing consumption of junk food (food that is ultra-processed, highly energy-dense, and rich in fat, salt, and glycemic load) (20–23), declining physical activity and increasing favor of a sedentary lifestyle (24, 25), rapid urbanization and increasing food accessibility (20, 26) jointly contribute to the surging prevalence of overweight and obesity in China. However, the overall prevalence of overweight and obesity could also be affected by population aging, since the prevalence of overweight/obesity increases with age (4, 17). China has been undergoing a rapid aging transition during the past three decades. The old-age dependency ratio (ratio of people ≥65 to people aged between 15 and 64) increased sharply from 8.35% in 1990 to 17.80% in 2019 (**Figure S1**). Therefore, because the prevalence of overweight and obesity varies greatly over different age-sex cohorts, the projected overweight/obesity epidemic could be biased in previous studies that assumed constant population distribution (1, 4, 16, 17). In particular, the aging population can further exacerbate the rising prevalence of overweight and obesity in China, which is not well addressed in previous literature.

To address this research gap, we presented detailed analyses using data from 9 consecutive nationally representative health surveys conducted between 1991 and 2015. We adopted two different approaches to estimate the national prevalence of overweight/obesity. First, we estimated the national prevalence of overweight/obesity using 1990 constant population distribution; then we re-estimated these prevalence using the age and sex distributions in the yearly 0.1% national sample census. The differences between these two estimations could be attributable to population aging. Furthermore, we projected the population and overweight/obesity prevalence for each age-sex cohort from 2016 to 2030, and estimated the overall national prevalence of overweight/obesity for Chinese adults using two different methods during the same period.

## Methods

### Study Design

We obtained data from the recent nine waves of the China Health and Nutrition Survey (CHNS) (1991, 1993, 1997, 2000, 2004, 2006, 2009, 2011 and 2015). The CHNS is a large cross-sectional survey conducted serially over the year, which is jointly conducted by the National Institute for Nutrition and Health at the Chinese Center for Disease Control and Prevention (CCDC) and the Carolina Population Center at the University of North Carolina at Chapel Hill. The survey adopted a multistage, stratified, and random cluster sampling strategy in nine provinces of mainland China (Liaoning, Heilongjiang, Jiangsu, Shandong, Henan, Hubei, Hunan, Guangxi, and Guizhou, three mega cities, Beijing, Chongqing, and Shanghai, joined the survey in 2011) to select approximately 4000-5000 households in each wave. The CHNS collected comprehensive information on anthropometric measures such as weight, height, and WC for each individual in surveyed families. More information about the survey has been described elsewhere (27).

### Study Population

In this study, we focused on adults aged no younger than 20 years old. In total, we obtained 96541 observations, but 21487 of them did not have weight, height or WC information, which were eliminated in this study. Participants with missing physical examination information were slightly younger and more likely to from rural area (**Table S1**), but those differences were small and should not affect our main estimation. We further excluded women who were pregnant (n=340) because their nutritional status (overweight/obesity) could not be defined by BMI or WC. Moreover, to reduce measurement error and ensure that the variables of interest were biologically plausible, we followed suggestions in previous literature and eliminated observations whose height was below 50 cm or weight was less than 20 kg (7) and respondents with an uncorrected BMI of <15 or >45 and WC of > 120 cm or <50 cm (n=585) (1, 17). We sequentially excluded observations with no sex or age recorded (n=2679). Finally, 71450 observations were maintained in the study, which were derived from 23280 Chinese adults. The sample sizes were 5062 in 1991, 6511 in 1993, 7124 in 1997, 7947 in 2000, 7834 in 2004, 7668 in 2006, 8169 in 2009, 10826 in 2011, and 10309 in 2015 (**Figure S2**). The population was further classified into 6 age groups for both sexes: 20-29 years old, 30-39 years old, 40-49 years old, 50-59 years old, 60-69 years old, and equal to or older than 70 years old.

### Anthropometric Measurements and Classification

The weight and height of each individual in CHNS were measured by trained health workers using regularly calibrated equipment and according to the manufacturer’s instructions (SECA880 scales and SECA 206 wall-mounted metal tapes). Weight was recorded to the nearest 0.1 kg with lightweight clothing on a calibrated beam scale, and height (without shoes) was measured to the nearest 0.1 cm. WC was measured at a point midway between the lowest rib and the iliac crest in a horizontal plane using nonelastic tape. BMI was calculated as body weight (kg) divided by height (m) squared (kg/m^2^). Detailed information can be found in previous research (27).

We adopted the criteria of the Working Group on Obesity in China (WGOC) to define overweight and general obesity for Chinese adults: overweight (BMI≥24 kg/m^2^ and <28 kg/m^2^) and general obesity (BMI≥28 kg/m^2^) (28). Abdominal obesity was defined as an individual’s WC≥90 cm for men and ≥85 cm for women, respectively (17, 28).

### Estimating National Prevalence of Overweight/Obesity From 1990 to 2015 Using Two Different Approaches

As BMI and WC vary among people in different age-sex cohorts, the age and sex distribution of the sample became the key determinants of sample representativeness. To remove the bias, we adopted two different methods to estimate the national prevalence of overweight/obesity. The first method followed previous studies by adopting a constant population distribution at the base year to adjust the age and sex distribution in the sample and estimated national prevalence of overweight and obesity (1, 16, 17). In this study we used the age and sex distribution of the Population Census of China in 1990, which was available from the National Bureau of Statistics of China (NBSC). The second method adopted the age and sex distribution in the yearly 0.1% national sample censuses (1% in 2015 and 0.1% in other years) to adjust population distribution in our sample and estimate national prevalence of each BMI category and abdominal obesity, which allows the age and sex distributions vary in different years. The impact of the aging population on overweight/obesity estimation can be detected by comparing the two estimations using different population distributions as references.

### Association Between Age and Risk of Overweight/Obesity

A multivariable logistic regression was conducted to detect the association between age and the risk of being affected by overweight and general obesity/abdominal obesity among Chinese adults, where the outcome variable was defined as underweight and normal/without abdominal obesity (y=0), overweight and general/abdominal obesity (y=1). We take people aged between 20 and 29 as the reference group and used five binary variables to control the rest five age groups. In addition, the association between overweight/obesity risk and age was further demonstrated using a local polynomial smoothing plot with 95% confidence intervals.

### Projection Population Structure From 2016 to 2030

The population was further projected from 2020 to 2030 using Bayesian hierarchical modeling, which was used in the 2019 Revision of the World Population Prospects by the United Nations Population Division (UNPD) (29). We adopted the UNPD estimation of the medium-fertility scenario as our reference population. As UNPD only projected the future population for every 5-year interval (2020, 2025 and 2030), we adopted the linear interpolation method to estimate the age-sex distribution of the population for missing values in the remaining 8 years (2021-2024, 2026-2029) (**Figure S3**). The age-sex distribution of the population between 2016 and 2019 was drawn directly from the 0.1% national sample census, which was available from the NBSC website.

### Projecting Prevalence of Overweight/Obesity From 2016 to 2030 for Each Age-Sex Cohort

Age- and sex-specific overweight and obesity prevalence were projected using linear regression. We took the overweight/obesity risks in 9 CHNS waves (1991-2015) as outcome variables and time (set 1991 as 1) and time square as the two dependent variables and adopted a linear model to estimate an annual growth rate for each age-sex cohort. The estimated growth rate was further used to project the overweight/obesity risk for each age-sex cohort from 2016 to 2030.

### Projecting National Prevalence of Overweight/Obesity From 2016 to 2030

Finally, the overall prevalence of overweight/obesity in Chinese adults was projected from 2016 to 2030 using the aforementioned two approaches: the first one adopted the constant population in 2015, and the second one adopted the projected population distribution in each year between 2016 and 2030.

All empirical analyses were conducted in Stata MP 14.0 (Stata Corp., College Station, TX, USA). We adopted a P value < 0.05 as the cutoff point for significance.

## Results

### Secular Trends of BMI and WC From 1991 to 2015

**Figure 1:**
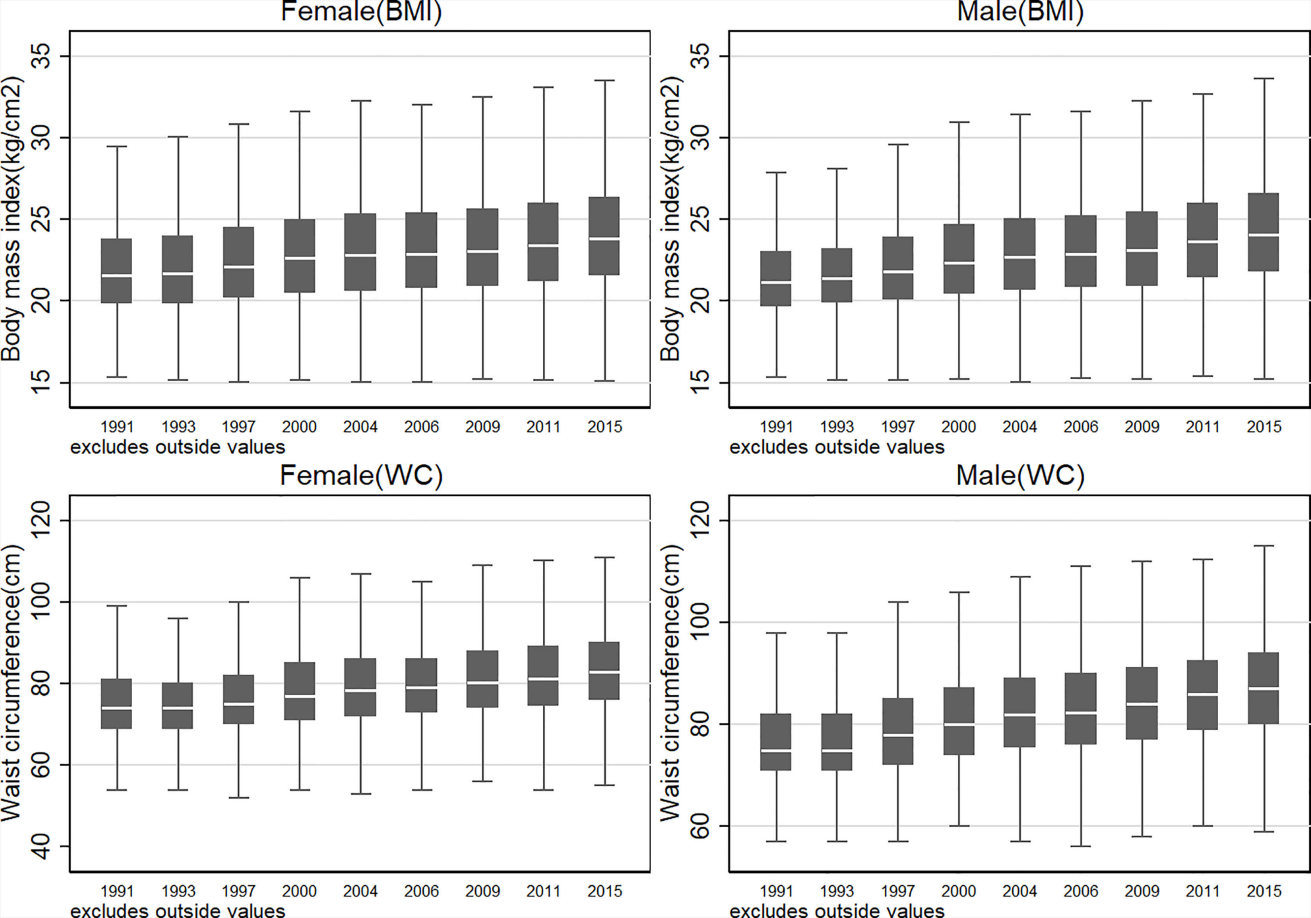
Secular trend of body mass index and waist circumference in Chinese adults from 1991 to 2015. The blank bar within the box refers to the median, the box refers to the upper (75^th^ percentile) and lower (25^th^ percentile) hinges, and the two solid short lines refer to upper and lower adjacent values.

**Figure 1** presents the secular trends of BMI and WC in Chinese adults from 1991 to 2015.During this period, the mean BMI and WC increased by 2.45 kg/m^2^ and 8.70 cm, respectively, and a greater increase was observed among men (2.79 kg/m^2^ and 10.08 cm) than among women (2.16 kg/m^2^ and 7.48 cm, respectively; more detailed information is shown in **Table 1**). Consequently, the kernel density distribution of both BMI and WC shifted gradually to the right for both women and men (**Figure S4**). Notably, the net increase in BMI and WC varied greatly across different age-sex cohorts. In particular, BMI grew more rapidly among young men and old women, and the largest BMI increase was observed among young men aged between 20-29 (3.29 kg/m^2^) and women older than or equal to 70 years old (3.08 kg/m^2^).

**Table 1:** Trends in mean body mass index and waist circumference among Chinese adults: 1991-2015.

	year	1991		1993		1997		2000		2004		2006		2009		2011		2015		Δ(%)	*P*trend
		n	Mean (SD)	n	Mean (SD)	n	Mean (SD)	n	Mean (SD)	n	Mean (SD)	n	Mean (SD)	n	Mean (SD)	n	Mean (SD)	n	Mean (SD)		
BMI (kg/m^2^)																					
Total		5062	21.78(2.84)	6511	21.91(2.85)	7124	22.38(3.12)	7947	22.85(3.24)	7834	23.08(3.32)	7668	23.21(3.31)	8169	23.37(3.45)	10826	23.80(3.55)	10309	24.23(3.71)	2.45(11.26%)	<0.01
Women		2697	21.99(3.03)	3411	22.09(3.02)	3668	22.52(3.25)	4167	22.95(3.35)	4131	23.17(3.47)	4071	23.27(3.43)	4284	23.39(3.51)	5753	23.76(3.68)	5472	24.15(3.72)	2.16(9.81%)	<0.01
age group	20~29	580	21.10(2.27)	734	21.02(2.19)	685	21.45(2.68)	608	21.40(2.75)	412	21.24(2.71)	338	21.21(2.84)	357	21.10(2.94)	490	21.62(3.53)	469	21.96(4.22)	0.86(4.09%)	<0.01
	30~39	734	21.91(2.64)	865	22.05(2.73)	822	22.56(2.93)	983	22.75(3.07)	813	22.76(3.22)	750	22.62(3.01)	658	22.69(3.29)	828	23.07(3.57)	656	23.21(3.59)	1.30(5.92%)	<0.01
	40~49	580	22.59(3.12)	755	22.82(3.08)	888	22.99(2.94)	1029	23.60(3.03)	981	23.58(3.22)	949	23.61(3.24)	989	23.82(3.27)	1337	24.06(3.47)	1098	24.30(3.45)	1.71(7.59%)	<0.01
	50~59	413	22.59(3.63)	492	22.57(3.39)	552	22.92(3.49)	693	23.74(3.49)	963	23.77(3.40)	985	23.92(3.26)	1051	24.05(3.27)	1391	24.55(3.58)	1278	24.76(3.51)	2.17(9.62%)	<0.01
	60~69	253	22.46(3.66)	359	22.63(3.65)	465	23.17(3.96)	516	23.25(3.86)	557	23.60(3.86)	592	23.89(3.74)	697	23.82(3.72)	1017	24.27(3.65)	1240	24.75(3.48)	2.29(10.19%)	<0.01
	>=70	137	20.97(2.97)	206	21.24(3.15)	256	21.60(3.73)	338	22.26(3.77)	405	22.90(4.03)	457	22.96(3.92)	532	23.15(3.92)	690	23.20(3.80)	731	24.06(3.93)	3.08(14.71%)	<0.01
Men		2365	21.54(2.60)	3100	21.72(2.63)	3456	22.23(2.98)	3780	22.73(3.12)	3703	22.99(3.14)	3597	23.13(3.18)	3885	23.34(3.38)	5073	23.84(3.40)	4837	24.33(3.71)	2.79(12.95%)	<0.01
age group	20~29	557	20.93(2.14)	715	21.06(2.27)	742	21.39(2.60)	635	21.80(2.96)	423	22.15(3.12)	338	22.56(3.31)	362	22.09(3.50)	446	23.06(3.95)	460	24.23(5.19)	3.29(15.74%)	<0.01
	30~39	581	21.64(2.41)	745	21.87(2.35)	747	22.51(2.74)	858	23.00(3.04)	694	23.00(3.11)	627	23.31(3.20)	612	23.66(3.46)	691	24.18(3.32)	538	24.70(4.22)	3.06(14.15%)	<0.01
	40~49	509	21.80(2.49)	685	22.08(2.56)	809	22.53(2.84)	880	23.03(2.94)	874	23.41(3.00)	842	23.57(3.02)	876	23.97(3.27)	1202	24.30(3.23)	947	24.61(3.36)	2.81(12.90%)	<0.01
	50~59	369	21.92(2.87)	425	22.06(2.84)	526	22.43(3.06)	692	22.94(3.00)	852	23.30(3.05)	885	23.30(3.00)	946	23.52(3.19)	1209	23.95(3.24)	1166	24.57(3.40)	2.64(12.06%)	<0.01
	60~69	256	21.71(3.29)	377	21.87(3.28)	401	22.59(3.60)	439	22.85(3.42)	505	22.86(3.19)	541	22.90(3.12)	656	23.28(3.25)	948	23.80(3.45)	1094	24.16(3.29)	2.45(11.29%)	<0.01
	>=70	93	21.11(2.84)	153	21.14(2.86)	231	21.93(3.39)	276	22.29(3.61)	355	22.41(3.44)	364	22.28(3.59)	433	22.40(3.53)	577	22.89(3.37)	632	23.50(3.55)	2.40(11.36%)	<0.01
Waist circumference (cm)																					
Total		5062	76.29(8.93)	6511	76.02(8.89)	7124	77.76(9.47)	7947	79.66(9.85)	7834	80.98(9.86)	7668	81.43(9.79)	8169	82.81(10.29)	10826	83.88(10.38)	10309	84.99(10.58)	8.70(11.40%)	<0.01
Women		2697	75.64(9.12)	3411	75.27(9.06)	3668	76.49(9.41)	4167	78.22(9.69)	4131	79.40(9.85)	4071	79.96(9.80)	4284	81.31(10.21)	5753	82.01(10.30)	5472	83.12(10.54)	7.48(9.88%)	<0.01
age group	20~29	580	72.04(7.56)	734	70.99(6.97)	685	72.27(7.68)	608	72.42(7.44)	412	72.81(7.46)	338	73.29(8.15)	357	73.75(8.77)	490	75.20(9.21)	469	75.53(10.55)	3.49(4.84%)	<0.01
	30~39	734	74.11(7.74)	865	73.85(7.86)	822	74.81(8.12)	983	75.79(8.42)	813	76.20(8.61)	750	76.36(8.28)	658	77.62(9.46)	828	78.32(9.10)	656	79.06(9.66)	4.96(6.69%)	<0.01
	40~49	580	77.18(8.70)	755	76.85(8.60)	888	77.38(8.36)	1029	79.17(8.63)	981	79.60(8.60)	949	79.51(8.92)	989	80.42(9.02)	1337	81.41(9.49)	1098	81.56(9.90)	4.38(5.67%)	<0.01
	50~59	413	78.79(10.08)	492	77.86(9.69)	552	78.63(9.62)	693	81.47(9.55)	963	81.65(9.37)	985	82.22(9.19)	1051	83.52(9.65)	1391	84.00(9.82)	1278	84.87(9.27)	6.08(7.71%)	<0.01
	60~69	253	79.34(10.10)	359	79.65(10.24)	465	80.60(11.08)	516	81.97(10.90)	557	82.91(10.95)	592	82.97(10.42)	697	84.39(10.44)	1017	85.19(10.55)	1240	85.82(10.06)	6.48(8.17%)	<0.01
	>=70	137	76.25(11.47)	206	76.84(10.71)	256	77.98(11.60)	338	80.38(11.35)	405	81.90(11.54)	457	82.94(10.95)	532	84.15(10.66)	690	83.74(10.83)	731	86.32(11.17)	10.07(13.20%)	<0.01
Men		2365	77.04(8.64)	3100	76.86(8.63)	3456	79.12(9.34)	3780	81.25(9.79)	3703	82.73(9.57)	3597	83.10(9.51)	3885	84.47(10.11)	5073	86.00(10.05)	4837	87.11(10.21)	10.08(13.08%)	<0.01
age group	20~29	557	74.70(7.46)	715	73.93(7.51)	742	75.81(8.15)	635	77.70(9.24)	423	79.90(9.42)	338	80.84(9.39)	362	79.91(10.11)	446	82.32(10.73)	460	84.23(10.80)	9.53(12.75%)	<0.01
	30~39	581	76.59(7.96)	745	76.42(7.84)	747	79.42(8.73)	858	81.57(9.56)	694	82.05(9.20)	627	82.35(9.25)	612	83.98(10.31)	691	86.23(9.97)	538	86.65(10.50)	10.06(13.14%)	<0.01
	40~49	509	77.61(8.29)	685	77.60(8.20)	809	79.84(8.58)	880	81.37(9.60)	874	83.43(9.09)	842	83.74(9.01)	876	85.42(9.80)	1202	87.01(9.80)	947	87.42(9.88)	9.82(12.65%)	<0.01
	50~59	369	78.51(9.02)	425	78.29(8.80)	526	79.82(9.58)	692	82.21(9.23)	852	83.52(9.56)	885	83.71(9.45)	946	85.20(9.72)	1209	86.49(9.81)	1166	88.11(9.83)	9.60(12.23%)	<0.01
	60~69	256	79.58(10.73)	377	79.44(10.44)	401	81.44(11.09)	439	83.18(10.14)	505	83.43(9.66)	541	83.26(9.57)	656	85.39(9.82)	948	86.37(9.86)	1094	87.28(9.99)	7.70(9.68%)	<0.01
	>=70	93	77.89(9.71)	153	79.05(9.76)	231	80.67(10.79)	276	82.62(11.07)	355	82.83(10.76)	364	83.34(10.79)	433	84.10(10.66)	577	84.87(10.21)	632	87.00(10.70)	9.11(11.70%)	<0.01

Value in brackets are standard deviation.

### Associations Between Age and the Risk of Being Affected by Overweight/Obesity

**Figure 2:**
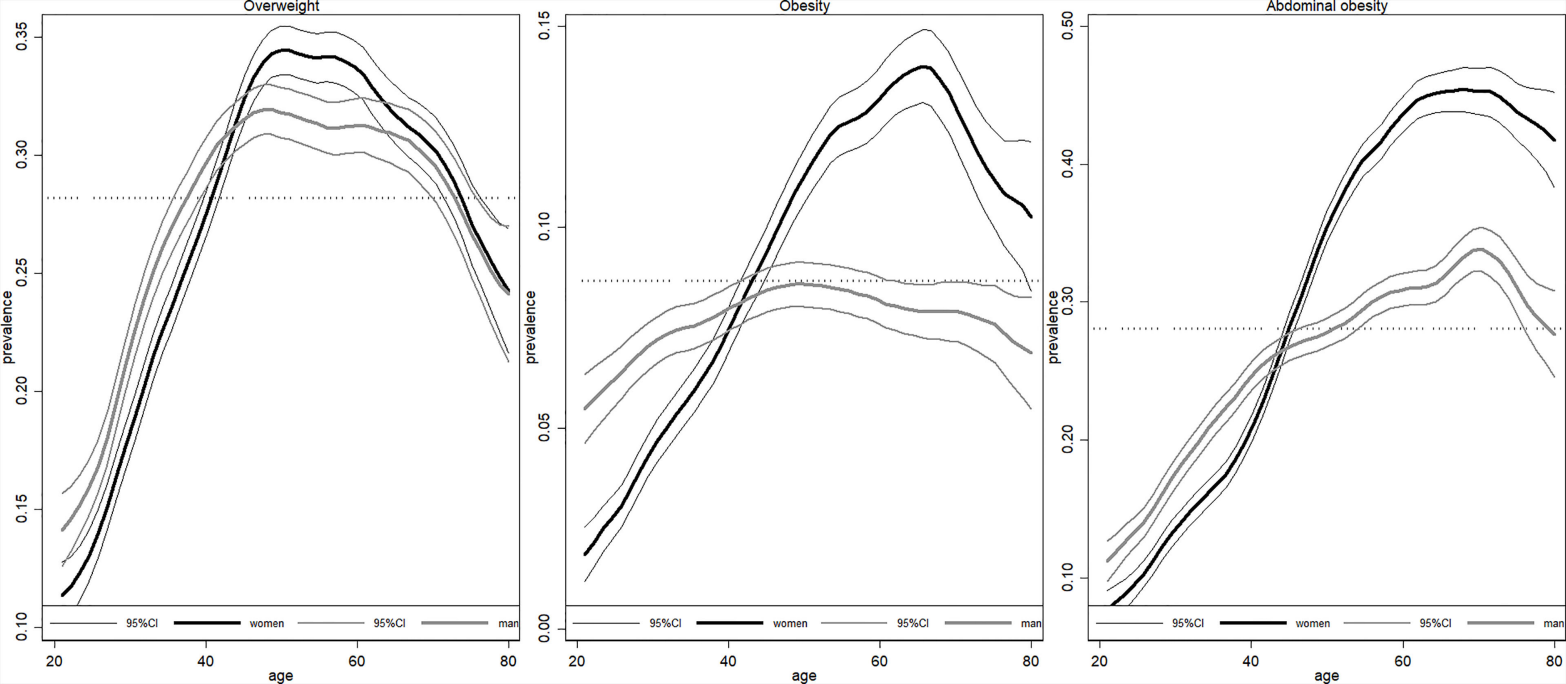
Association between age and risk of obesity/overweight. Figures are drawn using local polynomial regression. The black curve refers to women, and the gray curve refers to men. 95%CI refers to 95% confidence interval.

The prevalence of overweight, general obesity and abdominal obesity increased significantly for all age-sex cohorts during 1991-2015 (**Table S2**). **Figure 2** further demonstrated the association between overweight/obesity risk and age. We detected a very clear inverted U-shaped relationship for both women and men. The risk of being overweight peaked at approximately 50 years old for both women (black curve) and men (gray curve), but the top of the inverted U-shape was much flatter for men than women. Moreover, the general and abdominal obesity risk of women continued to grow until they were close to 70 years old. Additionally, compared with adults aged between 20 and 29, the odds of being affected by overweight/obesity peaked at approximately 50-59 years old in both women [OR=5.02, 95% CI (4.58,5.50)]men [OR=2.54, 95% CI (2.33,2.77)], and the odds of being affected by abdominal obesity was the highest at approximately 60-69 for both women [OR=7.86, 95% CI (7.03,8.78)] and men [OR=3.17, 95% CI (2.86,3.52)] (**Table 2**). Estimation after controlling other confounders generated similar results (**Table S3**).

**Table 2:** Association between age and risk of being affected by overweight/obesity.

	Overweight/obesity (logistic)						Abdominal obesity (logistic)					
	Women			Men			Women			Men		
	OR	CI_low	CI_up	OR	CI_low	CI_up	OR	CI_low	CI_up	OR	CI_low	CI_up
age20-29(ref)												
age30-39	2.32*	2.11	2.55	1.96*	1.79	2.14	1.89*	1.68	2.12	1.83*	1.65	2.04
age40-49	3.99*	3.65	4.38	2.53*	2.33	2.75	3.61*	3.23	4.02	2.49*	2.26	2.76
age50-59	5.02*	4.58	5.50	2.54*	2.33	2.77	6.41*	5.75	7.14	2.87*	2.60	3.17
age60-69	4.86*	4.41	5.35	2.43*	2.22	2.66	7.86*	7.03	8.78	3.17*	2.86	3.52
age70+	3.20*	2.88	3.55	1.83*	1.65	2.03	7.23*	6.43	8.14	3.08*	2.74	3.45
Obs.	37654			33796			37654			33796		
Pseudo R^2^	0.0383			0.0148			0.0673			0.0192		
chi^2^	1895.75*			650.91*			3087.22*			737.20*		

*Refers to statistical significance at 5%. CI refers to 95% confidence interval.

### Using a Consistent Population Structure Generally Underestimates the Prevalence of Obesity and Overweight

**Figure 3:**
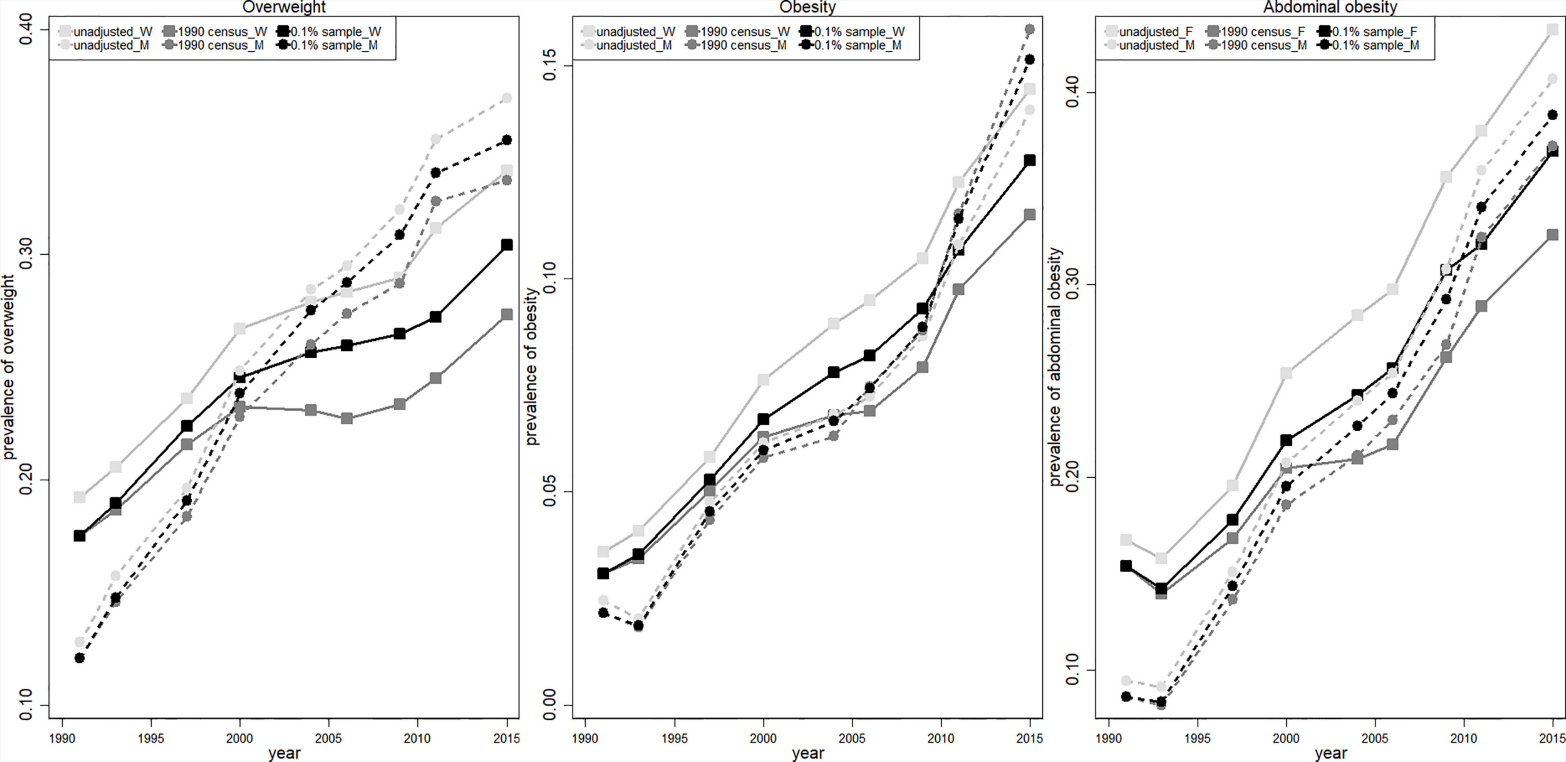
Comparison of three overweight/obesity estimations from 1991 to 2015. The light gray curve (unadjusted) is calculated using population structure in CHNS sample; the dark gray curve (1990 census) refers to the adjusted value using constant population structure in 1990 census; the black curve (0.1% sample) refers to the adjusted value using yearly 0.1% national sample census. Women and men are presented in solid (squared marker) and dashed (circle marker) curves, respectively.

The crude prevalence of overweight and obesity between 1991 and 2015 (light gray curve) is presented in **Figure 3**, together with two age-sex standardized estimations (values are presented in **Table S4**). All three curves showed a significant increase in the prevalence of overweight and obesity. More importantly, the overweight, general obesity and abdominal obesity prevalence adjusted by the 1990 census population (dark gray curve) was generally lower than that adjusted by the yearly 0.1% national sample census (black curve), and the gap enlarged over time. In contrast, the prevalence of general obesity in men (dashed curve with circle marker) estimated by the three methods was very similar. The disparities between the two estimations using different reference populations were attributable to population aging between 1991 and 2015. Our results indicated that ignoring the aging effect will lead to 3.07/4.34 and 1.77/1.63 percentage-point (pp) underestimation of overweight/abdominal obesity prevalence among women and men in 2015, respectively. In addition, ignoring the aging population led to an underestimation of general obesity among women by 1.27 pp and an overestimation of general obesity among men by 0.71 pp in 2015.

### Projection of Overweight/Obesity Between 2016 and 2030 With a Dynamic Population Structure

The projected risks of overweight and obesity for most age-sex cohorts will generally keep increasing from 2016 to 2030, which could be reflected by the upward secular trends (**Table S5** and **Figure S5**). In addition, the overweight occurrence of middle aged women (≥50 and <59) and men (≥40 and <49), as well as the obesity risk of both old women (≥70) and men (≥70) presented an inverted-U trend, which increased in the near future but turned to decrease in the long run (**Table S5** and **Figure S5**).

**Figure 4:**
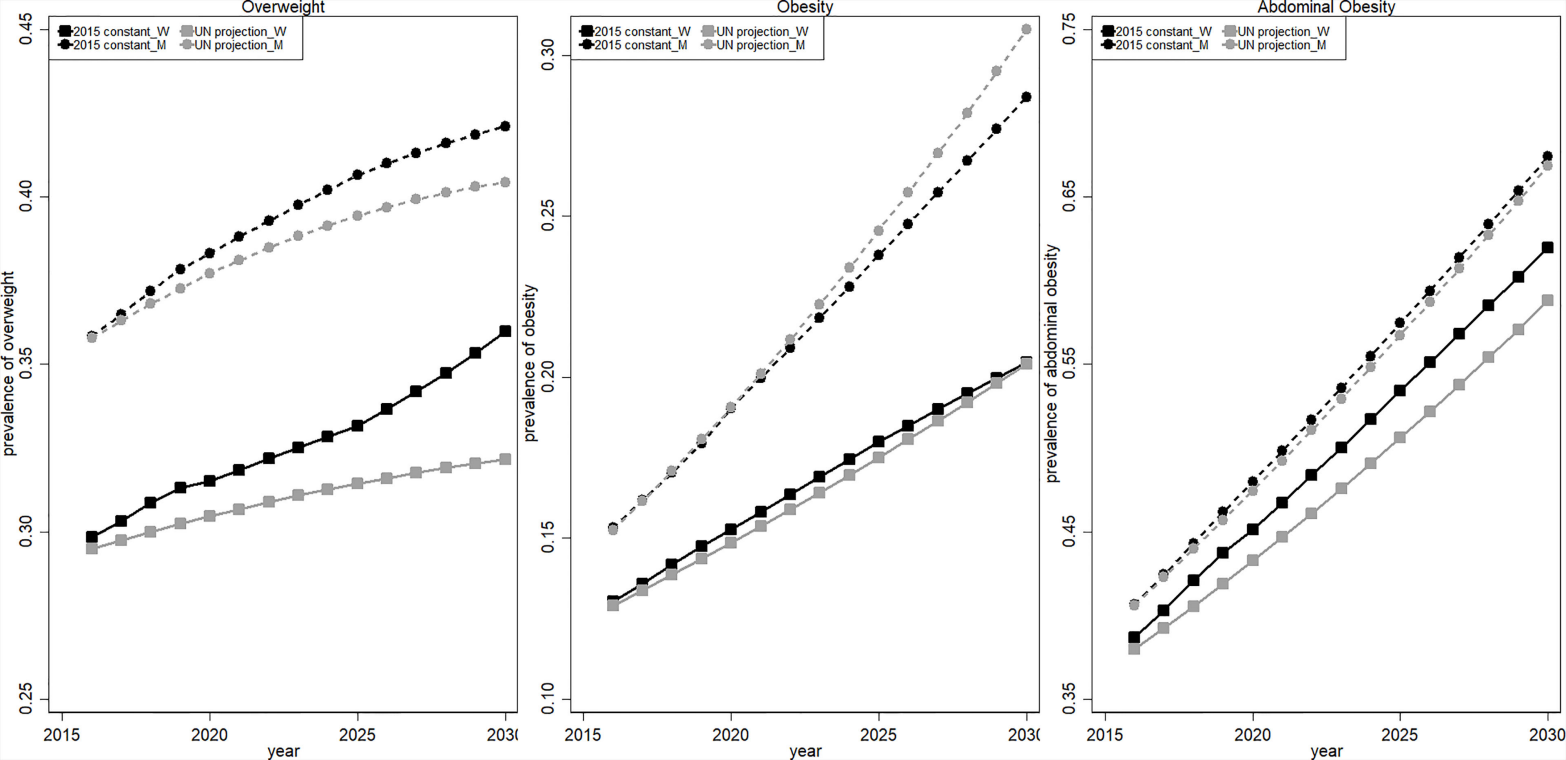
Projected overweight/obesity prevalence between 2016 and 2030. The black curve (2015 constant) is calculated using constant population structure in the 2015 1% sample census; the gray curve (UN projection) refers to the adjusted value using yearly projected population structure by UN. Women and men are presented in solid (squared marker) and dashed (circle marker) curves, respectively.

The projected overall prevalence of overweight/obesity among Chinese adults from 2016 to 2030 is presented in **Figure 4**. The projected prevalence of overweight using the yearly UNPD population projection (black line) increased slightly to 35.99% and 42.10% in 2030 among women (solid line with circle marker) and men (dashed line with square marker), respectively, but the prevalence of general obesity grew quickly to 20.45% and 28.70% among women and men in 2030. The largest increase was observed in the prevalence of abdominal obesity. Our projection indicated that more than 60% of women and men will suffer from abdominal obesity in 2030. By comparing the two estimations with or without taking population aging into account, we found that ignoring population aging (using 2015 1% national sample census data to standardize) will underestimate the prevalence of overweight, general obesity and abdominal obesity for women by 3.81, 0.06, and 3.16 pp (gray line), respectively. Meanwhile, the prevalence of overweight and abdominal obesity among men will be underestimated by 1.67 and 0.53 pp, respectively, but the prevalence of general obesity among men will be overestimated by 2.11 pp.

## Discussion

In the present study, 71450 observations were extracted from CHNS data between 1991 and 2015 to demonstrate the secular trend of national prevalence of overweight, general obesity and abdominal obesity in Chinese adults. We proposed two different approaches to adjust the age and sex distribution in the sample. The first one employed the population at the base year and assume a constant population distribution through the whole period, and the second one adopted the yearly 0.1% national sample census. The impact of population aging on the estimation of overweight and obesity was detected by comparing the estimations from two methods. Our results indicated that ignoring population aging would underestimate the prevalence of overweight by 3.07 and 1.77 pp in 2015 for women and men, respectively, and the prevalence of abdominal obesity by 4.34 and 1.63 pp for women and men, respectively. In addition, ignoring population would lead to an underestimation of general obesity by 1.27 pp in women but overestimation of general obesity by 0.71 pp in men. We further projected the national prevalence of overweight and obesity for each age-sex cohort from 2016 to 2030 using the same approaches: the first one employed constant population in 2015 and the second one adopted varying population between 2016 and 2030, which was estimated using the three 5-year-interval population projections (2020, 2025, 2030) proposed by UNPD. Finally, the results indicated that the prevalence of overweight will increase slightly to 35.99% and 42.10% in 2030 among women and men, respectively, but the prevalence of general obesity will grow quickly to 20.45% and 28.70% among women and men in 2030. The most striking result is that more than 60% of Chinese adults will suffer from abdominal obesity in 2030.

The present study is consistent with previous research, and the increased prevalence of overweight and obesity in Chinese adults is indisputable (30). Pan et al. used five Chinese national-level survey datasets to analyze BMI trends since the 1980s and found that the prevalence of overweight and obesity gradually increased with age (30). The prevalence of overweight and obesity was 31.9% and 11.6% in those aged 60 years and older, respectively, in 2012 (30). Notably, the prevalence of abdominal obesity increased faster than that of general obesity, particularly in women aged 60-69. Previous research demonstrated that the U.S. Men and women aged 60 years and older were more likely to be affected by obesity than their counterparts aged younger than 50 (31). Our results showed that Chinese women who were above 60 years old were twice as likely to be affected by obesity than their counterparts who were younger than 40. Along with economic development and improving the healthcare system, life expectancy at birth has been and will continue to increase for both sexes. Therefore, an aging population and a growing prevalence of obesity will trigger more disease burdens, such as hypertension, sarcopenia and type 2 diabetes mellitus (32). In addition, a current study using the 2011-2012 National Health and Nutrition Examination Survey (NHANES) also found a significant increase in obesity among individuals aged 60 years and older in the United States, but the prevalence of overweight and obesity did not increase very fast within young adults (33). However, our study detected a significantly increased risk of overweight/obesity for all age-sex cohorts over time, which might indicate that more comprehensive prevention strategies should be designed across the board. In countries where the population structure is stable (e.g., the U.S.), estimating and projecting the national prevalence of obesity using a constant population structure at the base period would not cause large bias. However, in countries that are undergoing a rapidly aging population such as China, assuming a constant population structure will significantly underestimate the national prevalence of obesity since older people are more likely to be affected by obesity. According to the National Bulletin on the Development of Aging, the share of elderly people (≥65) increased rapidly from 5.6% in 1990 to 13.5% in 2020, and China had more than 1.9 billion people older than 65 years old in 2020, which ranked China as the country with the largest number of older populations worldwide (34, 35). The aging population further exacerbated the rising prevalence of overweight and obesity in China, which could threaten the public health system.

We also noticed that there were a certain number of populations whose weight or BMI was in the normal range, while their waist circumference was larger than the recommended values, particularly in women. Extremely high abdominal obesity among old adults is of great concern, as abdominal obesity is considered to be more strongly associated with various noncommunicable diseases, such as type 2 diabetes, hypertension, dyslipidemia, metabolic syndrome, cardiovascular disease, cancer, and the risk of obesity-related morbidity and mortality, than general obesity (36–38). Moreover, women have shown a higher prevalence of abdominal obesity, and their risk of abdominal obesity continues to grow until they are 70 years old. One possible reason was that those women are experiencing the transition of menopause, since the average menopausal age of Asian women is 50 years old (39). After menopause, with the decline of estrogen, fat will mainly accumulate in the abdomen and viscera (40). With the increased lifespan, women spend almost one-third of their lifetime after menopause, which is considered a risk factor for obesity and obesity-related CVD and type 2 diabetes (41).

Furthermore, we also noted that as more adults cross the threshold to obesity, the growth rate of overweight prevalence slowed down in the future, while the prevalence of general obesity and abdominal obesity accelerated at an increasing rate. In total, approximately 2 in 3 Chinese adults will suffer from overweight or obesity in 2030, of which 20.45% and 28.70% of women and men will be affected by obesity, respectively. The predicted prevalence of abdominal obesity exceeds 50% for all age-sex cohorts except young females (≥30 & <39), and the share of adults with abdominal obesity will account for 64.71% of total Chinese adults in 2030. In addition, men were more vulnerable to general obesity and overweight than women. The increasing occurrence of general obesity among men was mainly driven by young men aged between 20 and 29, whose general obesity risk will reach 42.58% in 2030, while their overweight risk will increase slightly from 24.73% in 2016 to 26.43% in 2030. In contrast, women aged between 50 and 59 will experience the most rapid increase in general obesity prevalence, while their overweight prevalence will decline by approximately 13 pp during 2016-2030. The surging prevalence of general obesity and abdominal obesity suggests that they will become the most common adiposity categories among Chinese adults in the near future. Previous studies denoted that obesity is associated with more serious negative health consequences and disease burden compared with overweight, such as a higher rate of chronic disease (42), shorter life expectancy (11), larger deaths and disability-adjusted life years (9).

Previous studies found that the increasing prevalence of overweight and obesity could be attributable to declining physical activity (24), improving economic conditions, increasing consumption of high-calorie density food (23), shrinking household size and changing family structure (25), improving sanitation conditions (43) and urbanization. Similar results were also found in our study (**Tables S3**, **S6**, **S7** and **Figure S6**). It is technically much easier and cheaper to produce, distribute and sell energy-dense and nutrient-poor processed food with a long shelf duration than fresh healthy food (44). Therefore, we need to rethink our food system and develop various cost-effective prevention approaches to promote the production, distribution and consumption of healthy food. For instance, dietary education, school nutrition improvement programs, fitness equipment construction in neighborhoods, taxing unhealthy food, food labeling with nutrient information, regular physical examination and body mass monitoring are urgently needed to promote healthy lifestyles and prevent further weight gain. However, the effectiveness of various policies should be analyzed using appropriate methodology and data.

This study has several strengths. First, it included a large sample size (>70000 observations) from diverse and representative regions across China over more than 20 years, which allowed sufficient statistical power to investigate the secular trend of overweight and obesity prevalence among various age-sex cohorts. Second, it adjusted the population distribution using two different methods, and the one using yearly 0.1% national sample census took population aging into account. Thus the impact of population aging on obesity estimation could be investigated by comparing the estimations from two methods. Third, the prevalence of overweight and obesity was further projected to 2030 based on an age-sex cohort prediction and population projection.

This study, however, was also subjected to several limitations. First, the prediction of overweight and obesity prevalence for each age-sex cohort was based on a simple linear regression, which may overestimate the prevalence if the upward trend slowed down in the future. Second, the disease burden caused by population aging and the increasing prevalence of overweight and obesity were not estimated due to data availability. Economic analysis of the loss due to population aging and obesity epidemics can provide a more intuitive way to show the seriousness of the situation and contribute to the development of more effective and urgent approaches to prevent the epidemic. Third, many other factors such as urbanization, socio-economic factors and health literacy level may also contribute to the rising prevalence of overweight/obesity. **Tables S6** and **S7** presented the prevalence of overweight/obesity for each age-gender cohort by area, and we did found some differences between rural and urban areas. In addition, **Figure S6** presented the association between age and the risk of being affected by overweight/obesity for both rural and urban areas. Even some differences have been detected, the main conclusion would not change. Therefore, future studies with more detailed national population data (e.g., age-gender cohort population by area) can investigate the impact of other factors such as urbanization on overweight/obesity.

## Conclusion

The present study illustrated that ignoring population aging would significantly underestimate the national occurrence of overweight/obesity among China adults due to higher risk of overweight/obesity in old people. Our projection showed that the occurrence of overweight and general obesity will be greater than 55% and 70% in women and men respectively, and more than 60% of women and men will suffer from abdominal obesity in 2030. Our findings indicate that policy makers should design more precise prevention measurements for men and women at different ages and promote healthy life style in publics to reduce the risk of obesity.

## Data Availability Statement

The original contributions presented in the study are included in the article/**Supplementary Material**. Further inquiries can be directed to the corresponding author.

## Ethics Statement

The CHNS data are public data from an observational study that is not a clinical trial study. This survey was approved by the Institutional Review Boards of the University of North Carolina at Chapel Hill and the National Institute for Nutrition and Food Safety and China Center for Disease Control and Prevention. The patients/participants provided their written informed consent to participate in this study.

## Author Contributions

XT analyzed the data, drafted the initial manuscript and revised it. HW designed the study, analyzed the data and comprehensively revised the manuscript. All authors contributed to the article and approved the submitted version.

## Funding

The study was sponsored by the Discipline Construction Funding of Public Health and Preventive Medicine from the Peking University Health Science Center (BMU2021YJ046) and the Chinese Universities Scientific Fund from China Agricultural University.

## Conflict of Interest

The authors declare that the research was conducted in the absence of any commercial or financial relationships that could be construed as a potential conflict of interest.

## Publisher’s Note

All claims expressed in this article are solely those of the authors and do not necessarily represent those of their affiliated organizations, or those of the publisher, the editors and the reviewers. Any product that may be evaluated in this article, or claim that may be made by its manufacturer, is not guaranteed or endorsed by the publisher.
