# Supplementary material for: Projecting National-Level Prevalence of General Obesity and Abdominal Obesity Among Chinese Adults With Aging Effects
Source: Front Endocrinol (Lausanne). 2022 Mar 8;13:849392. doi: 10.3389/fendo.2022.849392 (PMC8957832; doi:10.3389/fendo.2022.849392)
Supplement: Supplementary file 1 [file DataSheet_1.docx]

Table S1: Age and region distribution of included and excluded samples

|  | Included | | | Excluded | | |
| --- | --- | --- | --- | --- | --- | --- |
|  | total | urban | rural | total | urban | rural |
| age | 48.04 | 49.16 | 47.42 | 42.79 | 44.56 | 42.08 |
| sample | 75054 | 26766 | 48288 | 21324 | 7011 | 14313 |
| proportion | 77.87% | 79.24% | 77.14% | 22.13% | 20.76% | 22.86% |

Note: Included refers to sample used in present study, and excluded refers to sample not used in this study due to missing information on physical examination.

Table S2 Secular trend of overweight/obesity for each age-sex cohort

| Overweight | Women | | | | | | Men | | | | | |
| --- | --- | --- | --- | --- | --- | --- | --- | --- | --- | --- | --- | --- |
| Year | 20~29 | 30~39 | 40~49 | 50~59 | 60~69 | >=70 | 20~29 | 30~39 | 40~49 | 50~59 | 60~69 | >=70 |
| 1991 | 9.48% | 19.48% | 26.72% | 21.31% | 22.53% | 14.60% | 7.36% | 14.11% | 14.93% | 15.72% | 12.89% | 12.90% |
| 1993 | 10.22% | 19.31% | 29.01% | 23.37% | 26.46% | 14.56% | 7.69% | 16.78% | 20.00% | 18.82% | 17.51% | 15.69% |
| 1997 | 13.72% | 23.48% | 28.83% | 29.35% | 25.16% | 17.19% | 10.92% | 21.95% | 23.11% | 22.24% | 22.19% | 17.32% |
| 2000 | 12.66% | 24.31% | 32.75% | 35.21% | 27.52% | 21.89% | 14.49% | 26.22% | 29.77% | 26.45% | 26.65% | 21.74% |
| 2004 | 12.14% | 23.74% | 32.82% | 34.27% | 28.01% | 25.19% | 19.15% | 27.38% | 32.49% | 31.46% | 29.11% | 23.38% |
| 2006 | 10.65% | 23.73% | 31.93% | 34.52% | 32.09% | 23.41% | 22.19% | 27.43% | 33.73% | 32.54% | 29.76% | 22.25% |
| 2009 | 12.32% | 23.71% | 29.63% | 35.87% | 32.28% | 27.63% | 18.78% | 32.52% | 37.67% | 32.88% | 33.99% | 25.87% |
| 2011 | 12.45% | 22.83% | 34.26% | 38.39% | 36.18% | 26.52% | 23.99% | 36.18% | 38.27% | 37.30% | 36.29% | 29.29% |
| 2015 | 15.57% | 26.98% | 36.43% | 36.70% | 38.87% | 33.65% | 23.91% | 36.25% | 39.18% | 39.71% | 39.67% | 33.86% |
| obesity | Women | | | | | | Men | | | | | |
| year | 20~29 | 30~39 | 40~49 | 50~59 | 60~69 | >=70 | 20~29 | 30~39 | 40~49 | 50~59 | 60~69 | >=70 |
| 1991 | 0.52% | 1.77% | 3.79% | 8.72% | 8.30% | 1.46% | 0.72% | 1.72% | 1.96% | 4.34% | 6.64% | 1.08% |
| 1993 | 0.27% | 2.89% | 5.03% | 7.52% | 8.36% | 3.40% | 1.12% | 0.94% | 2.04% | 3.53% | 4.51% | 1.31% |
| 1997 | 2.04% | 5.11% | 4.95% | 7.43% | 12.04% | 6.25% | 2.70% | 4.15% | 4.70% | 5.32% | 7.48% | 7.36% |
| 2000 | 2.47% | 5.49% | 8.75% | 10.82% | 11.43% | 7.40% | 4.25% | 6.88% | 5.68% | 5.92% | 8.20% | 6.88% |
| 2004 | 2.43% | 5.90% | 8.77% | 11.01% | 13.11% | 11.36% | 4.73% | 7.06% | 7.32% | 7.75% | 5.94% | 6.48% |
| 2006 | 2.37% | 5.47% | 9.17% | 11.88% | 13.85% | 11.16% | 7.40% | 8.29% | 8.19% | 6.78% | 4.99% | 7.42% |
| 2009 | 3.36% | 7.14% | 11.12% | 12.75% | 12.77% | 10.71% | 7.18% | 10.95% | 10.84% | 8.56% | 6.86% | 5.08% |
| 2011 | 5.51% | 8.94% | 11.89% | 15.53% | 14.75% | 11.45% | 10.99% | 13.60% | 13.14% | 9.68% | 9.49% | 6.76% |
| 2015 | 7.68% | 9.60% | 13.48% | 17.37% | 17.42% | 14.36% | 17.39% | 17.29% | 15.31% | 15.18% | 10.88% | 9.65% |
| Abdominal obesity | Women | | | | | | Men | | | | | |
| year | 20~29 | 30~39 | 40~49 | 50~59 | 60~69 | >=70 | 20~29 | 30~39 | 40~49 | 50~59 | 60~69 | >=70 |
| 1991 | 7.41% | 10.08% | 18.62% | 28.09% | 31.23% | 22.63% | 3.41% | 7.40% | 9.43% | 14.09% | 17.19% | 18.28% |
| 1993 | 4.77% | 9.83% | 18.15% | 25.00% | 31.20% | 22.33% | 3.36% | 6.71% | 8.91% | 12.94% | 17.51% | 17.65% |
| 1997 | 7.88% | 12.29% | 19.59% | 25.72% | 36.77% | 29.69% | 7.14% | 13.79% | 15.20% | 18.06% | 23.94% | 22.51% |
| 2000 | 6.41% | 15.77% | 26.63% | 38.24% | 39.15% | 36.69% | 10.55% | 20.86% | 21.36% | 21.53% | 28.02% | 28.26% |
| 2004 | 7.04% | 15.50% | 25.99% | 38.11% | 41.11% | 41.48% | 15.37% | 20.17% | 25.06% | 27.58% | 25.54% | 27.89% |
| 2006 | 8.58% | 15.87% | 26.66% | 38.17% | 40.20% | 43.11% | 19.53% | 20.26% | 26.72% | 28.25% | 25.51% | 29.67% |
| 2009 | 12.04% | 20.97% | 29.73% | 44.81% | 47.49% | 46.62% | 16.57% | 30.72% | 32.76% | 32.35% | 33.84% | 30.95% |
| 2011 | 15.71% | 22.83% | 34.48% | 44.72% | 50.54% | 46.67% | 23.32% | 35.46% | 40.18% | 35.73% | 39.03% | 32.58% |
| 2015 | 19.19% | 26.83% | 35.15% | 49.92% | 54.92% | 54.17% | 29.57% | 38.66% | 41.71% | 43.65% | 41.96% | 41.14% |

Notes: Prevalence is calculated based on the 1991-2015 CHNS sample.

Table S3 Potential risk factors for being affected by overweight/obesity

|  | overweight/obesity (female) | | | abdominal obesity (female) | | | overweight/obesity (male) | | | abdominal obesity (male) | | |
| --- | --- | --- | --- | --- | --- | --- | --- | --- | --- | --- | --- | --- |
|  | Coef. | 95% CI | | Coef. | 95% CI | | Coef. | 95% CI | | Coef. | 95% CI | |
| 30~39 | 2.85* | 2.46 | 3.30 | 1.88* | 1.60 | 2.21 | 2.37* | 2.05 | 2.73 | 2.15* | 1.83 | 2.52 |
| 40~49 | 5.27* | 4.57 | 6.08 | 3.55* | 3.04 | 4.13 | 2.93* | 2.56 | 3.34 | 2.59* | 2.23 | 3.00 |
| 50~59 | 6.19* | 5.33 | 7.20 | 5.84* | 4.99 | 6.84 | 2.52* | 2.20 | 2.89 | 2.73* | 2.35 | 3.17 |
| 60~69 | 4.70* | 3.96 | 5.57 | 6.62* | 5.57 | 7.88 | 2.29* | 1.96 | 2.67 | 2.76* | 2.34 | 3.26 |
| 70~ | 3.12* | 2.51 | 3.88 | 6.25* | 5.05 | 7.73 | 1.66* | 1.33 | 2.09 | 2.55* | 2.03 | 3.22 |
| education | 0.97* | 0.96 | 0.98 | 0.97* | 0.96 | 0.98 | 1.03* | 1.01 | 1.04 | 1.02* | 1.01 | 1.04 |
| minority | 1.06 | 0.95 | 1.17 | 0.83* | 0.75 | 0.93 | 0.71* | 0.63 | 0.80 | 0.68* | 0.60 | 0.78 |
| north | 1.25* | 1.16 | 1.34 | 1.30* | 1.21 | 1.39 | 1.42* | 1.32 | 1.53 | 1.76* | 1.62 | 1.90 |
| urban | 1.10* | 1.02 | 1.19 | 0.96 | 0.89 | 1.04 | 0.86* | 0.79 | 0.93 | 0.84* | 0.78 | 0.92 |
| activity | 1.04* | 1.01 | 1.08 | 1.02 | 0.98 | 1.05 | 0.86* | 0.83 | 0.90 | 0.83* | 0.80 | 0.87 |
| ln(calorie) | 1.27* | 1.13 | 1.42 | 1.16* | 1.05 | 1.30 | 1.53* | 1.36 | 1.72 | 1.24* | 1.10 | 1.40 |
| Fat_share | 1.17 | 0.85 | 1.61 | 1.04 | 0.76 | 1.42 | 1.39 | 0.99 | 1.96 | 1.83* | 1.28 | 2.60 |
| Protein_share | 2.89 | 0.84 | 9.98 | 1.10 | 0.33 | 3.69 | 21.24* | 5.77 | 78.27 | 22.08* | 5.88 | 82.89 |
| FAFH_share | 0.67* | 0.55 | 0.82 | 0.81* | 0.66 | 0.99 | 1.29* | 1.08 | 1.54 | 1.11 | 0.92 | 1.33 |
| smoking | 0.75* | 0.64 | 0.90 | 0.99 | 0.85 | 1.16 | 0.75* | 0.70 | 0.81 | 0.90* | 0.83 | 0.97 |
| alcohol | 0.99 | 0.96 | 1.02 | 1.01 | 0.98 | 1.04 | 0.94* | 0.92 | 0.97 | 0.95* | 0.93 | 0.97 |
| hhsize | 0.79* | 0.77 | 0.81 | 0.86* | 0.83 | 0.88 | 0.83* | 0.80 | 0.85 | 0.87* | 0.84 | 0.90 |
| ln(incomeper) | 1.00 | 0.97 | 1.02 | 0.97* | 0.95 | 1.00 | 1.04* | 1.01 | 1.06 | 1.03* | 1.01 | 1.06 |
| Child_share | 2.89* | 2.30 | 3.63 | 1.82* | 1.45 | 2.29 | 1.87* | 1.46 | 2.39 | 1.06 | 0.81 | 1.38 |
| Old_share | 1.36* | 1.14 | 1.61 | 1.38* | 1.17 | 1.61 | 0.97 | 0.80 | 1.19 | 1.17 | 0.96 | 1.43 |
| Max_bmi | 1.74* | 1.72 | 1.77 | 1.42* | 1.40 | 1.44 | 1.61* | 1.59 | 1.64 | 1.39* | 1.37 | 1.41 |
| Tap water | 1.14* | 1.04 | 1.25 | 1.05 | 0.97 | 1.15 | 0.99 | 0.90 | 1.09 | 1.01 | 0.91 | 1.13 |
| toilet | 0.98 | 0.89 | 1.06 | 1.00 | 0.92 | 1.09 | 1.03 | 0.94 | 1.12 | 1.04 | 0.95 | 1.14 |
| sanitation | 0.95 | 0.87 | 1.03 | 1.03 | 0.95 | 1.12 | 1.07 | 0.98 | 1.18 | 1.08 | 0.97 | 1.19 |
| Time | 0.98* | 0.96 | 1.00 | 0.99 | 0.96 | 1.01 | 1.05* | 1.03 | 1.08 | 1.03* | 1.00 | 1.06 |
| Time squared | 1.00 | 1.00 | 1.00 | 1.00* | 1.00 | 1.00 | 1.00* | 1.00 | 1.00 | 1.00 | 1.00 | 1.00 |
| Constant | 0.00* | 0.00 | 0.00 | 0.00* | 0.00 | 0.00 | 0.00* | 0.00 | 0.00 | 0.00* | 0.00 | 0.00 |
| Observations | 25,875 | | | 25,875 | | | 23,073 | | | 23,073 | | |
| Pseudo R^2^ | 0.337 | | | 0.242 | | | 0.315 | | | 0.244 | | |

Note: * refers to statistical significance at 5%. CI refers to confidence interval. A multivariable logistic regression was conducted to detect the association between age and the risk of being affected by overweight/obesity among Chinese adults, where the outcome variable was defined as underweight/normal (y=0), overweight and general obesity/ abdominal obesity (y=1). We take people aged between 20 and 29 as the reference group. In addition, individual characteristics (education attainment, minority, household registration location), physical activity, total daily calorie intake, share of calories drawn from fat (fat_share) and protein (protein_share), frequency of food away from home (FAFH), smoking (whether participants still smoke cigarette) and drinking (frequency of alcohol consumption) status, household characteristics (household size, household income per capita, share of children and older people), and genetic factors (maximum adult BMI in the family) were taken as confounders and controlled in the regression. Moreover, three binary variables were generated to measure the sanitary conditions of households and controlled in the regression as suggested by previous literature: access to clean drinking water (in-house or in-yard tap water), ownership of private toilets (in-house flush or in-house toilet) and basic sanitation services for disposing garbage (no near house excreta removal). Finally, to control the secular trend of overweight/obesity, time and time squared were controlled.

Table S4: Prevalence of obesity using different methods

| Methods | Year | Overweight | | Obesity | | Abdominal obesity | |
| --- | --- | --- | --- | --- | --- | --- | --- |
|  |  | Female | Male | Female | Male | Female | Male |
| Original value | 1991 | 19.21% | 12.77% | 3.60% | 2.45% | 16.72% | 9.43% |
|  | 1993 | 20.55% | 15.71% | 4.08% | 2.03% | 15.77% | 9.13% |
|  | 1997 | 23.61% | 19.62% | 5.81% | 4.75% | 19.57% | 15.10% |
|  | 2000 | 26.71% | 24.84% | 7.63% | 6.14% | 25.41% | 20.74% |
|  | 2004 | 27.91% | 28.44% | 8.93% | 6.81% | 28.42% | 23.95% |
|  | 2006 | 28.35% | 29.50% | 9.48% | 7.23% | 29.77% | 25.41% |
|  | 2009 | 28.99% | 31.99% | 10.48% | 8.65% | 35.60% | 30.81% |
|  | 2011 | 31.17% | 35.11% | 12.25% | 10.78% | 37.98% | 35.92% |
|  | 2015 | 33.75% | 36.94% | 14.44% | 13.95% | 43.26% | 40.67% |
| Constant structure in 1990 census | 1991 | 17.51% | 12.07% | 3.08% | 2.16% | 15.37% | 8.59% |
|  | 1993 | 18.67% | 14.59% | 3.44% | 1.83% | 13.96% | 8.17% |
|  | 1997 | 21.58% | 18.36% | 5.03% | 4.34% | 16.84% | 13.63% |
|  | 2000 | 23.26% | 22.81% | 6.29% | 5.80% | 20.47% | 18.57% |
|  | 2004 | 23.09% | 26.01% | 6.80% | 6.30% | 20.96% | 21.15% |
|  | 2006 | 22.73% | 27.37% | 6.89% | 7.47% | 21.69% | 22.98% |
|  | 2009 | 23.36% | 28.72% | 7.92% | 8.78% | 26.22% | 26.88% |
|  | 2011 | 24.50% | 32.36% | 9.74% | 11.51% | 28.88% | 32.45% |
|  | 2015 | 27.34% | 33.30% | 11.50% | 15.84% | 32.58% | 37.20% |
| Yearly 0.1% national sample census | 1991 | 17.51% | 12.07% | 3.08% | 2.16% | 15.37% | 8.59% |
|  | 1993 | 18.98% | 14.76% | 3.53% | 1.86% | 14.23% | 8.35% |
|  | 1997 | 22.38% | 19.06% | 5.29% | 4.53% | 17.79% | 14.34% |
|  | 2000 | 24.53% | 23.83% | 6.70% | 5.98% | 21.91% | 19.54% |
|  | 2004 | 25.66% | 27.53% | 7.79% | 6.65% | 24.26% | 22.64% |
|  | 2006 | 25.98% | 28.75% | 8.20% | 7.42% | 25.68% | 24.34% |
|  | 2009 | 26.47% | 30.86% | 9.30% | 8.86% | 30.75% | 29.23% |
|  | 2011 | 27.21% | 33.61% | 10.66% | 11.39% | 32.11% | 34.04% |
|  | 2015 | 30.41% | 35.07% | 12.77% | 15.13% | 36.93% | 38.83% |

Notes: Prevalence is estimated from population structure in CHNS sample (original value), adjusted by constant population structure in 1990 census (1990 census), and by yearly 0.1% national sample census, respectively.

Table S5 Fitted model of obesity/overweight between 1991 and 2015 for each age-sex cohort

|  | obesity | | | | | | | | | | | |
| --- | --- | --- | --- | --- | --- | --- | --- | --- | --- | --- | --- | --- |
| Obesity | female | | | | | | male | | | | | |
|  | 20~29 | 30~39 | 40~49 | 50~59 | 60~69 | >=70 | 20~29 | 30~39 | 40~49 | 50~59 | 60~69 | >=70 |
| t | -0.05 | 0.28 | 0.37* | -0.01 | 0.33* | 0.82* | -0.14 | 0.25 | 0.27* | -0.14 | -0.22 | 0.60** |
|  | (0.68) | (0.05) | (0.01) | (0.96) | (0.04) | (0.00) | (0.47) | (0.14) | (0.01) | (0.41) | (0.38) | (0.05) |
| t squared | 0.01* | 0.00 | 0.00 | 0.02* | 0.00 | -0.01 | 0.03* | 0.02* | 0.01* | 0.02* | 0.01 | -0.01 |
|  | (0.03) | (0.90) | (0.81) | (0.02) | (1.00) | (0.08) | (0.01) | (0.04) | (0.00) | (0.02) | (0.15) | (0.26) |
| Constant | 0.81 | 2.06* | 3.51* | 7.99* | 8.15* | 0.92 | 1.45 | 1.16 | 1.59* | 4.53* | 6.71** | 1.06 |
|  | (0.13) | (0.01) | (0.00) | (0.00) | (0.00) | (0.09) | (0.08) | (0.16) | (0.00) | (0.00) | (0.00) | (0.41) |
| Observations | 9 | 9 | 9 | 9 | 9 | 9 | 9 | 9 | 9 | 9 | 9 | 9 |
| F test | 43.66 | 61.15 | 242.51 | 63.77 | 31.53 | 112.54 | 72.33 | 202.80 | 341.45 | 38.74 | 5.45 | 10.07 |
| p | 0.00 | 0.00 | 0.00 | 0.00 | 0.00 | 0.00 | 0.00 | 0.00 | 0.00 | 0.00 | 0.04 | 0.01 |
| R^2^ | 0.91 | 0.92 | 0.96 | 0.94 | 0.89 | 0.96 | 0.96 | 0.97 | 0.99 | 0.93 | 0.52 | 0.65 |
|  | overweight | | | | | | | | | | | |
| Overweight | female | | | | | | male | | | | | |
|  | 20~29 | 30~39 | 40~49 | 50~59 | 60~69 | >=70 | 20~29 | 30~39 | 40~49 | 50~59 | 60~69 | >=70 |
| t | 0.09 | 0.42* | 0.31 | 1.66* | 0.16 | 0.63* | 1.18* | 1.23* | 1.80* | 1.29* | 1.37* | 0.51* |
|  | (0.72) | (0.09) | (0.30) | (0.00) | (0.57) | (0.04) | (0.00) | (0.00) | (0.00) | (0.00) | (0.00) | (0.09) |
| t squared | 0.00 | -0.01 | 0.00 | -0.04* | 0.02* | 0.01 | -0.02 | -0.01 | -0.03* | -0.01* | -0.01 | 0.01 |
|  | (0.82) | (0.47) | (1.00) | (0.01) | (0.06) | (0.64) | (0.13) | (0.23) | (0.00) | (0.03) | (0.15) | (0.28) |
| Constant | 10.46* | 19.34* | 27.41* | 19.64* | 23.75* | 13.48* | 4.96* | 13.32* | 13.65* | 14.61* | 12.72* | 13.43* |
|  | (0.00) | (0.00) | (0.00) | (0.00) | (0.00) | (0.00) | (0.01) | (0.00) | (0.00) | (0.00) | (0.00) | (0.00) |
| Observations | 9 | 9 | 9 | 9 | 9 | 9 | 9 | 9 | 9 | 9 | 9 | 9 |
| F test | 2.78 | 11.62 | 9.23 | 160.70 | 115.49 | 75.12 | 79.47 | 178.97 | 309.63 | 433.34 | 356.87 | 146.62 |
| p | 0.14 | 0.01 | 0.01 | 0.00 | 0.00 | 0.00 | 0.00 | 0.00 | 0.00 | 0.00 | 0.00 | 0.00 |
| R^2^ | 0.44 | 0.67 | 0.69 | 0.95 | 0.94 | 0.95 | 0.94 | 0.97 | 0.99 | 0.99 | 0.99 | 0.96 |
|  | Abdominal obesity | | | | | | | | | | | |
| Overweight | female | | | | | | male | | | | | |
|  | 20~29 | 30~39 | 40~49 | 50~59 | 60~69 | >=70 | 20~29 | 30~39 | 40~49 | 50~59 | 60~69 | >=70 |
| t | -0.52* | 0.18 | 0.56* | 0.81 | 0.49 | 1.70* | 0.67* | 0.92* | 1.18* | 0.65* | 0.45 | 0.63* |
|  | (0.02) | (0.39) | (0.07) | (0.14) | (0.10) | (0.01) | (0.05) | (0.09) | (0.01) | (0.02) | (0.35 | (0.09) |
| t squared | 0.04* | 0.02* | 0.01 | 0.01 | 0.02* | -0.01 | 0.02 | 0.02 | 0.01 | 0.02* | 0.02 | 0.01 |
|  | (0.00) | (0.04) | (0.50) | (0.64) | (0.09) | (0.19) | (0.13) | (0.36) | (0.36) | (0.02) | (0.26) | (0.54) |
| Constant | 7.49* | 9.83* | 17.21* | 24.57* | 30.78* | 19.44* | 2.11* | 5.85* | 6.91* | 12.38* | 17.35* | 17.43* |
|  | (0.52) | (0.18) | (0.56) | (0.81) | (0.49) | (1.70) | (0.67) | (0.92) | (1.18) | (0.65) | (0.45) | (0.63) |
| Observations | 9 | 9 | 9 | 9 | 9 | 9 | 9 | 9 | 9 | 9 | 9 | 9 |
| F test | 66.27 | 303.77 | 71.57 | 39.47 | 240.50 | 136.86 | 186.66 | 121.62 | 114.79 | 501.82 | 78.32 | 63.75 |
| p | 0.00 | 0.00 | 0.00 | 0.00 | 0.00 | 0.00 | 0.00 | 0.00 | 0.00 | 0.00 | 0.00 | 0.00 |
| R^2^ | 0.94 | 0.96 | 0.93 | 0.90 | 0.96 | 0.98 | 0.96 | 0.95 | 0.98 | 0.99 | 0.90 | 0.93 |

Notes: Prevalence was estimated using the linear model by regressing the overweight/obesity risks in 9 CHNS waves (1991-2015) on time (set 1991 as 1) and time square.

Table S6 Secular trend of overweight/obesity for each age-sex cohort in rural area

| Overweight | Women | | | | | | Men | | | | | |
| --- | --- | --- | --- | --- | --- | --- | --- | --- | --- | --- | --- | --- |
| Year | 20~29 | 30~39 | 40~49 | 50~59 | 60~69 | >=70 | 20~29 | 30~39 | 40~49 | 50~59 | 60~69 | >=70 |
| 1991 | 9.11% | 16.19% | 24.29% | 16.17% | 20.99% | 15.56% | 7.44% | 14.21% | 12.78% | 13.33% | 8.00% | 14.75% |
| 1993 | 11.02% | 16.58% | 26.02% | 18.46% | 20.17% | 13.39% | 6.24% | 15.03% | 16.98% | 17.51% | 12.23% | 14.58% |
| 1997 | 13.11% | 20.86% | 26.39% | 24.66% | 19.85% | 12.93% | 10.37% | 18.80% | 22.18% | 18.59% | 17.41% | 16.26% |
| 2000 | 12.41% | 23.91% | 29.47% | 31.22% | 20.13% | 21.57% | 14.42% | 24.36% | 28.87% | 25.00% | 22.26% | 21.05% |
| 2004 | 11.76% | 22.74% | 33.64% | 31.89% | 25.87% | 22.27% | 16.60% | 26.75% | 30.39% | 29.86% | 25.70% | 16.99% |
| 2006 | 12.02% | 24.86% | 30.71% | 32.92% | 29.63% | 21.61% | 18.96% | 28.77% | 33.57% | 29.53% | 26.20% | 18.54% |
| 2009 | 15.09% | 24.45% | 31.33% | 36.27% | 31.60% | 23.24% | 20.73% | 29.67% | 38.16% | 33.39% | 32.79% | 25.74% |
| 2011 | 15.75% | 23.55% | 35.20% | 38.75% | 34.06% | 24.74% | 25.00% | 34.23% | 39.13% | 35.07% | 32.72% | 28.14% |
| 2015 | 16.67% | 28.26% | 37.15% | 37.07% | 37.60% | 32.19% | 23.15% | 32.59% | 38.73% | 39.70% | 34.61% | 29.53% |
| Obesity | Women | | | | | | Men | | | | | |
| year | 20~29 | 30~39 | 40~49 | 50~59 | 60~69 | >=70 | 20~29 | 30~39 | 40~49 | 50~59 | 60~69 | >=70 |
| 1991 | 0.74% | 2.23% | 3.30% | 9.02% | 5.56% | 1.11% | 0.50% | 1.55% | 1.70% | 3.92% | 6.67% | 0.00% |
| 1993 | 0.39% | 3.76% | 4.83% | 6.15% | 8.82% | 2.36% | 1.32% | 1.00% | 1.68% | 3.37% | 3.49% | 1.04% |
| 1997 | 2.00% | 4.29% | 3.99% | 6.17% | 11.45% | 7.48% | 2.24% | 3.31% | 3.50% | 3.94% | 5.67% | 5.69% |
| 2000 | 2.63% | 6.02% | 8.36% | 9.17% | 10.74% | 5.88% | 3.78% | 6.47% | 4.12% | 4.09% | 6.57% | 5.26% |
| 2004 | 2.75% | 5.60% | 8.56% | 8.81% | 11.63% | 9.24% | 5.66% | 6.79% | 7.24% | 5.58% | 4.95% | 6.80% |
| 2006 | 1.92% | 5.31% | 9.72% | 11.59% | 12.43% | 9.16% | 7.11% | 8.45% | 6.96% | 6.39% | 4.79% | 4.88% |
| 2009 | 2.16% | 6.55% | 10.83% | 13.22% | 12.34% | 10.70% | 5.69% | 11.21% | 9.21% | 7.17% | 5.35% | 2.94% |
| 2011 | 8.06% | 8.88% | 12.05% | 16.25% | 12.82% | 8.85% | 9.77% | 15.16% | 12.38% | 9.58% | 7.95% | 5.09% |
| 2015 | 9.57% | 11.30% | 13.97% | 17.45% | 17.39% | 14.74% | 16.67% | 18.94% | 15.59% | 14.66% | 12.25% | 7.52% |
| Abdominal obesity | Women | | | | | | Men | | | | | |
| year | 20~29 | 30~39 | 40~49 | 50~59 | 60~69 | >=70 | 20~29 | 30~39 | 40~49 | 50~59 | 60~69 | >=70 |
| 1991 | 7.64% | 9.11% | 18.63% | 22.56% | 23.46% | 20.00% | 2.73% | 7.24% | 7.67% | 9.02% | 8.67% | 16.39% |
| 1993 | 4.92% | 9.06% | 17.29% | 19.38% | 23.53% | 19.69% | 2.27% | 6.61% | 7.34% | 10.10% | 10.48% | 12.50% |
| 1997 | 7.56% | 12.09% | 17.53% | 21.98% | 34.73% | 24.49% | 6.71% | 11.57% | 12.65% | 13.80% | 19.03% | 22.76% |
| 2000 | 6.92% | 16.09% | 26.54% | 34.50% | 33.22% | 32.35% | 9.69% | 20.61% | 19.76% | 17.46% | 22.99% | 23.39% |
| 2004 | 9.02% | 13.90% | 27.22% | 36.22% | 37.21% | 36.55% | 14.72% | 20.37% | 21.91% | 25.00% | 21.05% | 21.84% |
| 2006 | 8.65% | 14.99% | 25.62% | 38.64% | 37.04% | 42.12% | 18.01% | 22.15% | 25.18% | 26.25% | 22.54% | 22.93% |
| 2009 | 12.07% | 21.83% | 31.48% | 46.70% | 49.57% | 46.79% | 15.45% | 32.01% | 30.92% | 32.08% | 32.79% | 30.15% |
| 2011 | 18.68% | 24.38% | 37.83% | 47.25% | 49.92% | 46.35% | 23.05% | 36.92% | 39.39% | 34.79% | 34.38% | 29.34% |
| 2015 | 20.99% | 27.27% | 35.47% | 49.43% | 52.29% | 52.83% | 27.78% | 37.60% | 40.28% | 42.19% | 39.05% | 33.43% |

Notes: Prevalence is calculated based on the 1991-2015 CHNS sample.

Table S7 Secular trend of overweight/obesity for each age-sex cohort in urban area

| Overweight | Women | | | | | | Men | | | | | |
| --- | --- | --- | --- | --- | --- | --- | --- | --- | --- | --- | --- | --- |
| Year | 20~29 | 30~39 | 40~49 | 50~59 | 60~69 | >=70 | 20~29 | 30~39 | 40~49 | 50~59 | 60~69 | >=70 |
| 1991 | 10.34% | 26.25% | 33.33% | 30.61% | 25.27% | 12.77% | 7.14% | 13.92% | 19.75% | 21.05% | 19.81% | 9.38% |
| 1993 | 8.41% | 25.00% | 36.41% | 32.93% | 38.84% | 16.46% | 11.83% | 20.33% | 26.92% | 21.88% | 25.68% | 17.54% |
| 1997 | 14.89% | 27.83% | 33.33% | 39.11% | 32.02% | 22.94% | 12.00% | 27.76% | 24.75% | 29.82% | 29.87% | 18.52% |
| 2000 | 13.23% | 25.16% | 39.19% | 42.98% | 37.61% | 22.39% | 14.62% | 30.26% | 31.54% | 29.39% | 33.94% | 22.86% |
| 2004 | 12.74% | 25.87% | 31.19% | 38.64% | 31.46% | 29.34% | 23.42% | 28.85% | 36.36% | 34.46% | 35.16% | 32.21% |
| 2006 | 8.46% | 21.08% | 34.55% | 37.57% | 36.45% | 26.09% | 27.56% | 24.34% | 34.04% | 38.24% | 36.56% | 27.04% |
| 2009 | 7.20% | 22.00% | 25.82% | 35.14% | 33.62% | 34.63% | 14.66% | 39.13% | 36.57% | 31.93% | 36.28% | 26.09% |
| 2011 | 8.29% | 21.80% | 32.67% | 37.90% | 39.15% | 28.76% | 22.63% | 39.01% | 36.79% | 40.48% | 41.03% | 30.86% |
| 2015 | 13.10% | 24.90% | 35.08% | 36.11% | 40.76% | 35.49% | 25.74% | 43.58% | 40.13% | 39.73% | 47.17% | 39.56% |
| Obesity | Women | | | | | | Men | | | | | |
| year | 20~29 | 30~39 | 40~49 | 50~59 | 60~69 | >=70 | 20~29 | 30~39 | 40~49 | 50~59 | 60~69 | >=70 |
| 1991 | 0.00% | 0.83% | 5.13% | 8.16% | 13.19% | 2.13% | 1.30% | 2.06% | 2.55% | 5.26% | 6.60% | 3.13% |
| 1993 | 0.00% | 1.07% | 5.53% | 10.18% | 7.44% | 5.06% | 0.54% | 0.81% | 2.88% | 3.91% | 6.08% | 1.75% |
| 1997 | 2.13% | 6.47% | 6.73% | 10.06% | 12.81% | 4.59% | 3.60% | 5.70% | 6.78% | 8.19% | 10.39% | 9.26% |
| 2000 | 2.12% | 4.40% | 9.51% | 14.04% | 12.39% | 9.70% | 5.19% | 7.75% | 8.72% | 9.65% | 10.91% | 9.52% |
| 2004 | 1.91% | 6.56% | 9.17% | 15.04% | 15.49% | 14.37% | 3.16% | 7.69% | 7.47% | 11.82% | 7.69% | 6.04% |
| 2006 | 3.08% | 5.83% | 7.97% | 12.43% | 16.36% | 14.13% | 7.87% | 7.94% | 10.64% | 7.52% | 5.38% | 10.69% |
| 2009 | 5.60% | 8.50% | 11.76% | 11.89% | 13.62% | 10.73% | 10.34% | 10.33% | 14.55% | 11.14% | 9.73% | 8.70% |
| 2011 | 2.30% | 9.01% | 11.62% | 14.55% | 17.45% | 14.71% | 12.63% | 11.35% | 14.45% | 9.82% | 11.55% | 9.05% |
| 2015 | 3.45% | 6.83% | 12.57% | 17.24% | 17.47% | 13.89% | 19.12% | 13.97% | 14.72% | 16.03% | 8.84% | 12.45% |
| Abdominal obesity | Women | | | | | | Men | | | | | |
| year | 20~29 | 30~39 | 40~49 | 50~59 | 60~69 | >=70 | 20~29 | 30~39 | 40~49 | 50~59 | 60~69 | >=70 |
| 1991 | 6.90% | 12.08% | 18.59% | 38.10% | 45.05% | 27.66% | 5.19% | 7.73% | 13.38% | 25.44% | 29.25% | 21.88% |
| 1993 | 4.42% | 11.43% | 20.28% | 35.93% | 46.28% | 26.58% | 6.45% | 6.91% | 12.50% | 19.53% | 28.38% | 26.32% |
| 1997 | 8.51% | 12.62% | 23.40% | 33.52% | 39.41% | 36.70% | 8.00% | 17.87% | 19.66% | 26.90% | 31.82% | 22.22% |
| 2000 | 5.29% | 15.09% | 26.80% | 45.53% | 47.25% | 43.28% | 12.26% | 21.40% | 24.50% | 29.82% | 36.36% | 36.19% |
| 2004 | 3.82% | 18.92% | 23.55% | 41.59% | 47.42% | 48.50% | 16.46% | 19.71% | 30.84% | 32.43% | 33.52% | 36.24% |
| 2006 | 8.46% | 17.94% | 28.90% | 37.28% | 45.79% | 44.57% | 22.05% | 15.87% | 29.79% | 32.03% | 31.18% | 38.36% |
| 2009 | 12.00% | 19.00% | 25.82% | 41.35% | 43.40% | 46.34% | 18.97% | 27.72% | 36.94% | 32.83% | 35.84% | 32.30% |
| 2011 | 11.98% | 20.64% | 28.86% | 41.29% | 51.42% | 47.06% | 23.68% | 33.33% | 41.53% | 37.07% | 45.21% | 37.04% |
| 2015 | 15.17% | 26.10% | 34.55% | 50.71% | 58.84% | 55.86% | 33.82% | 40.78% | 44.82% | 46.05% | 46.26% | 51.28% |

Notes: Prevalence is calculated based on the 1991-2015 CHNS sample.


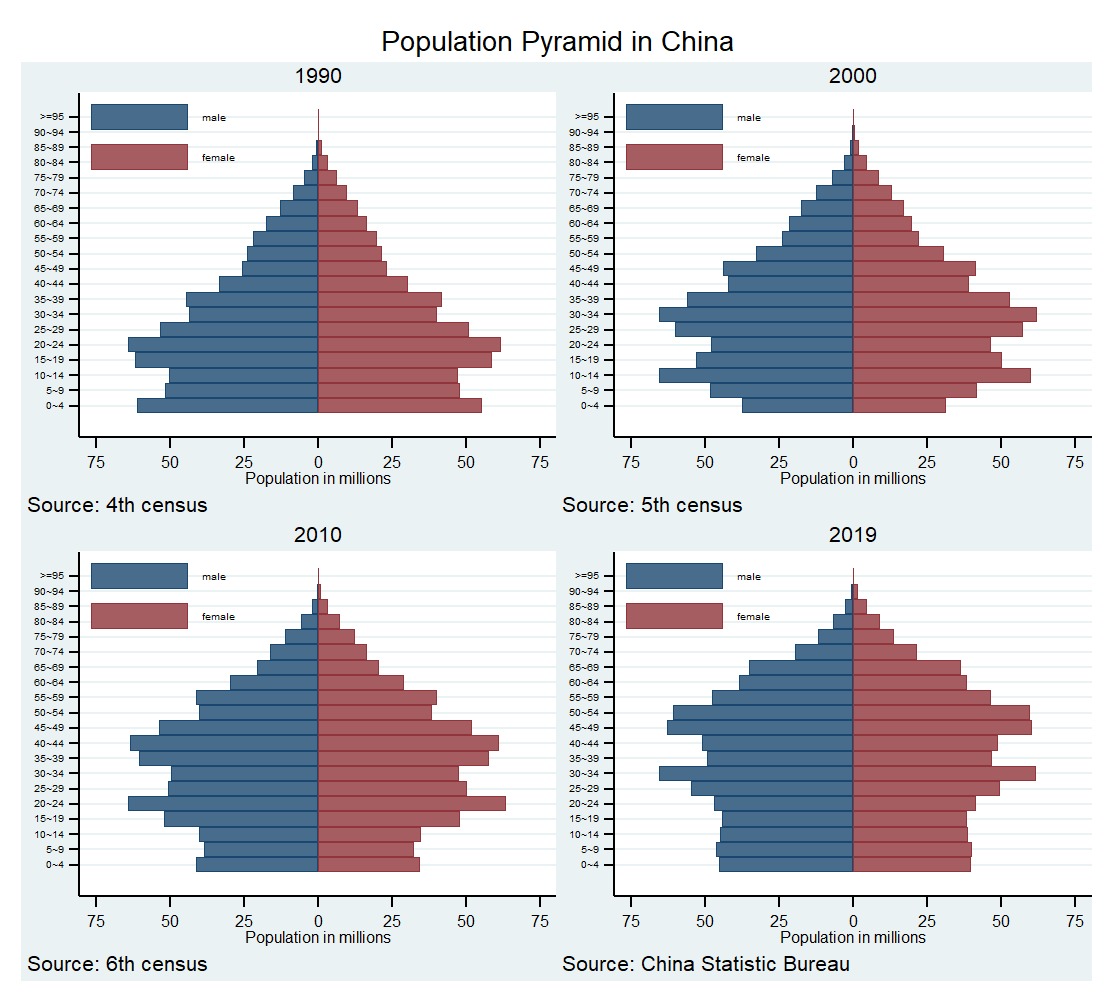
 Figure S1 Population pyramid between 1990 and 2019

Figure legend: Figures are based on the 4^th^, 5^th^, 6^th^ national census and the 0.1% national sample census in 2019.


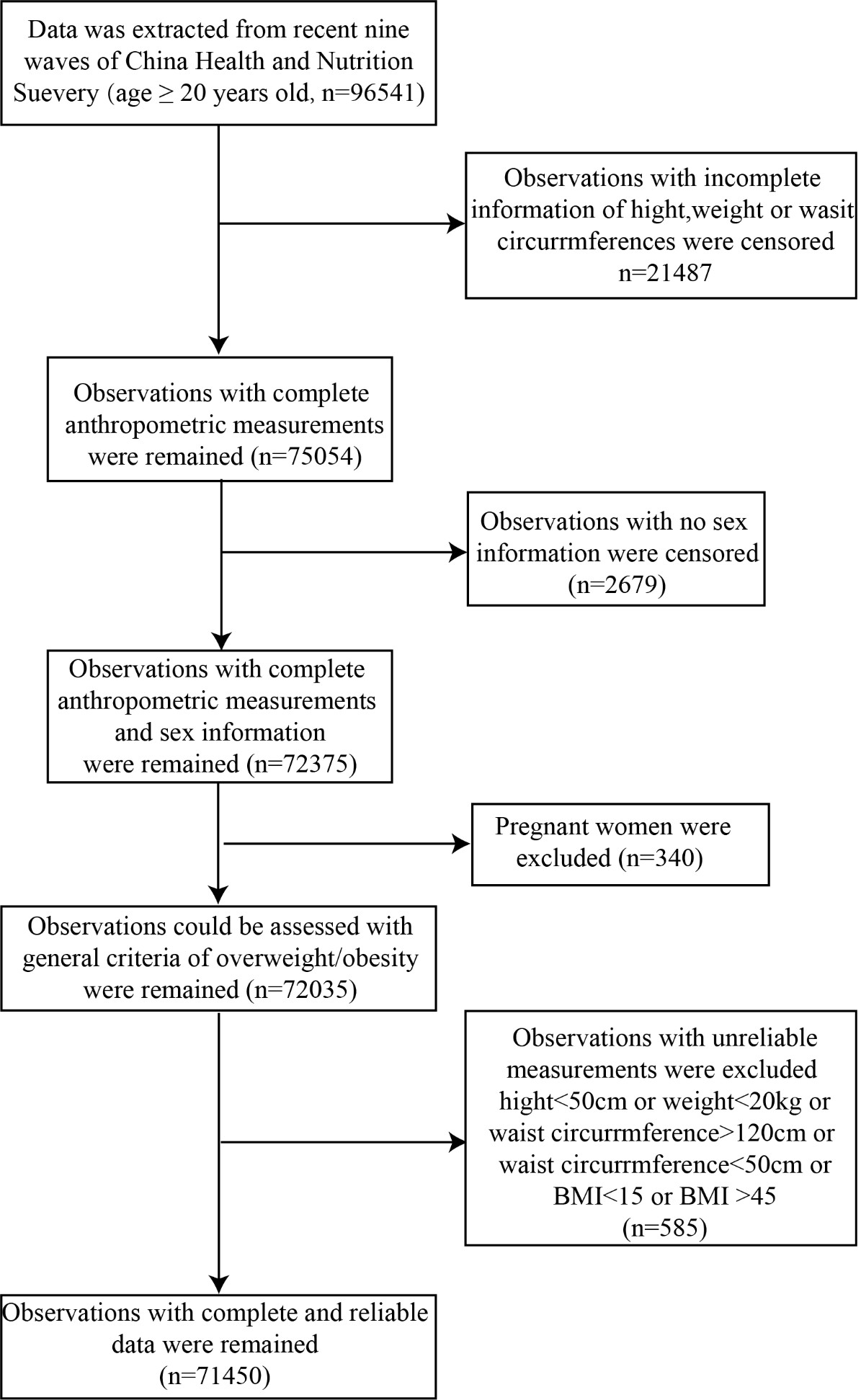


Figure S2 Flow chart


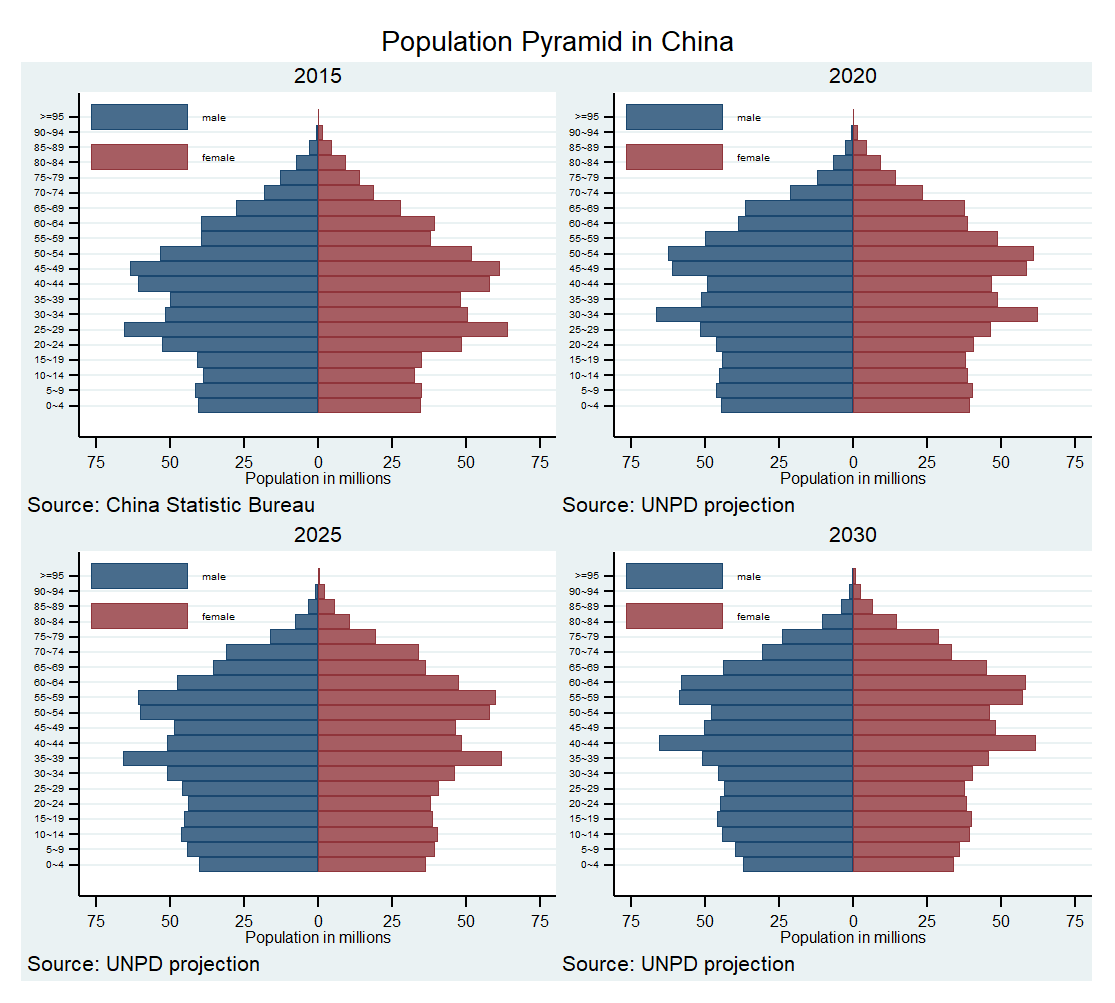


Figure S3: Population pyramid between 2015 and 2030

Figure legend: Figures are based on the population projection for China by UNPD.


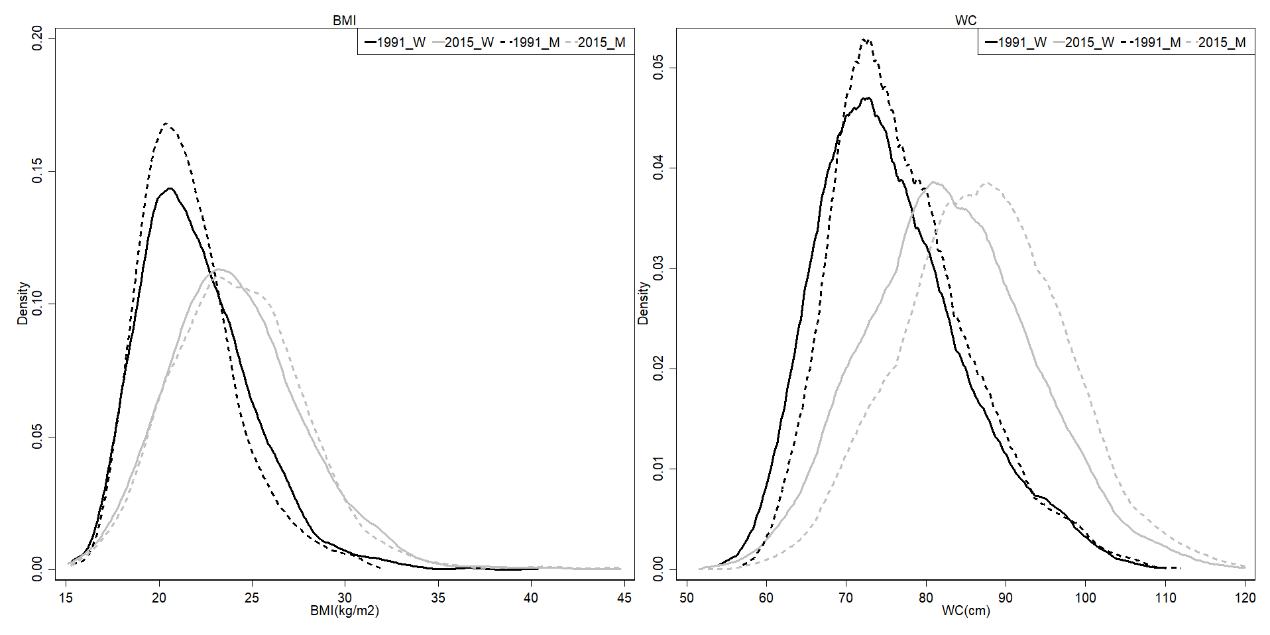


Figure S4: Distribution of body mass index and waist circumference in Chinese adults from 1991 to 2015

Legend: Data were drawn from adults aged 20 and above who participated in five waves of CHNS. The solid curve refers to women, and the dashed curve refers to men. Values in 1991 and 2015 are presented in black and gray, respectively.


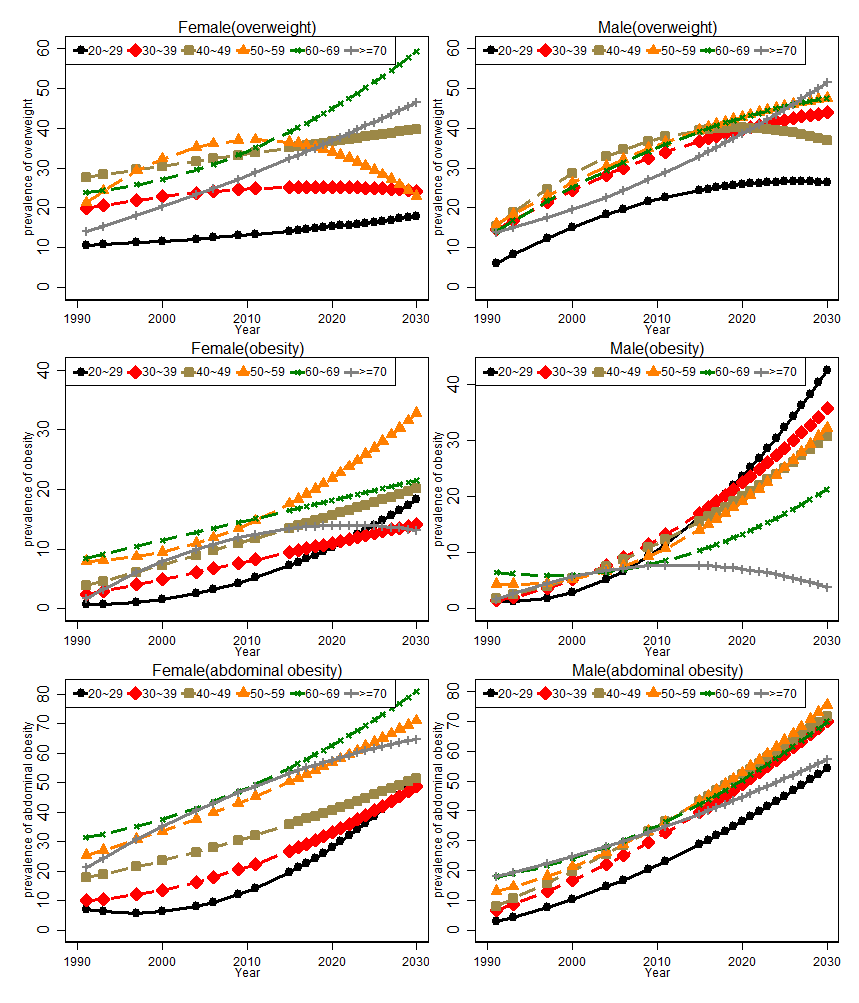


Figure S5: Projected obesity/overweight prevalence for each age cohort between 1990 and 2030

Figure legend: Value is predicted using a linear model by regressing observed prevalence at each age-sex cohort on time (set 1991 as 1) and time square.


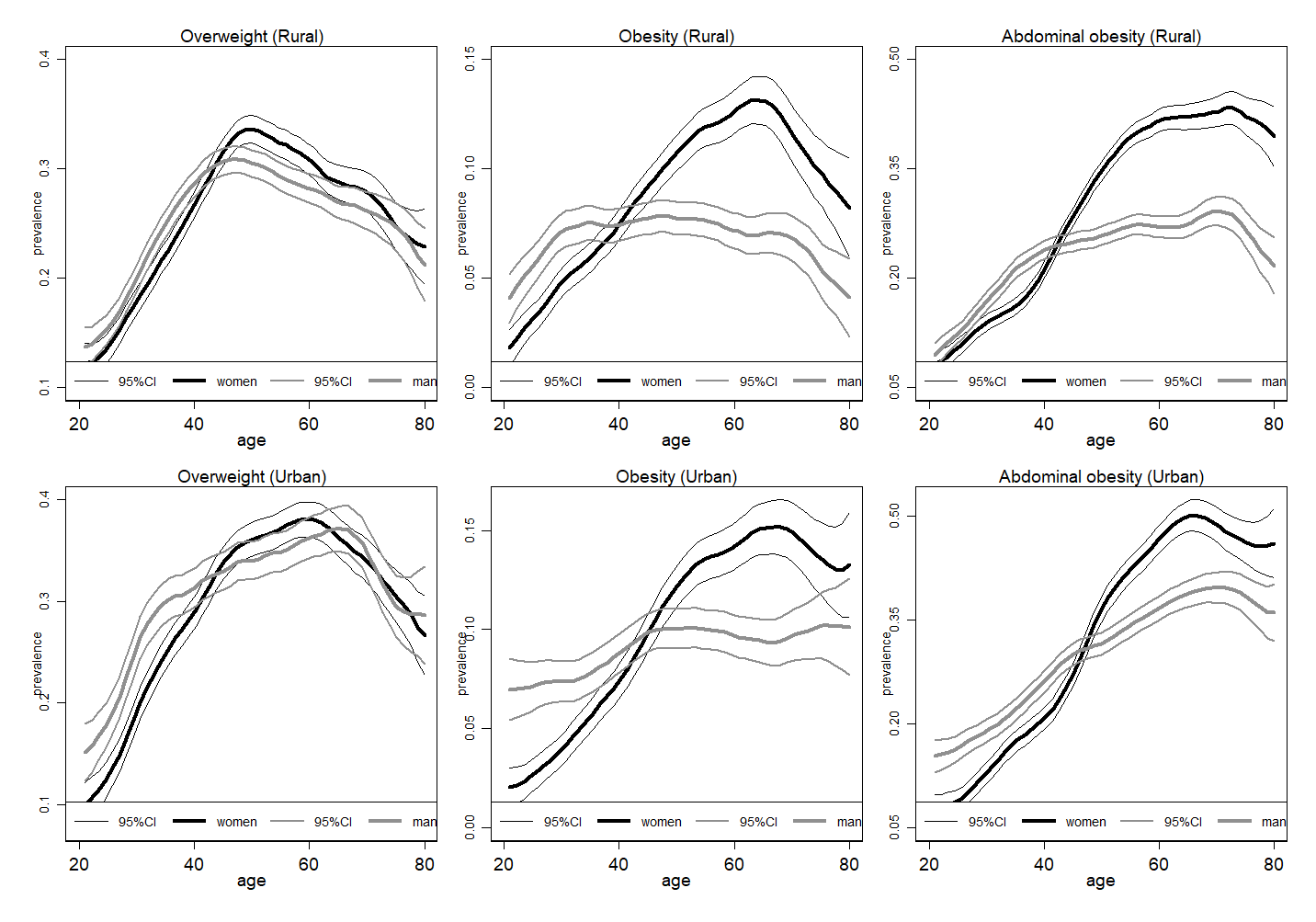


Figure S6: Association between age and risk of obesity/overweight by area

Legend: Figures are drawn using local polynomial regression. The black curve refers to women, and the gray curve refers to men. 95%CI refers to 95% confidence interval.
